# Associations of D-Dimer on Admission and Clinical Features of COVID-19 Patients: A Systematic Review, Meta-Analysis, and Meta-Regression

**DOI:** 10.3389/fimmu.2021.691249

**Published:** 2021-05-07

**Authors:** Runzhen Zhao, Zhenlei Su, Andrey A. Komissarov, Shan-Lu Liu, Guohua Yi, Steven Idell, Michael A. Matthay, Hong-Long Ji

**Affiliations:** ^1^ Department of Cellular and Molecular Biology, University of Texas Health Science Centre at Tyler, Tyler, TX, United States; ^2^ Department of Respiratory and Critical Care Medicine, Xinxiang Central Hospital, Xinxiang, Henan, China; ^3^ Texas Lung Injury Institute, The University of Texas Health Science Centre at Tyler, Tyler, TX, United States; ^4^ Department of Veterinary Biosciences, The Ohio State University, Columbus, OH, United States; ^5^ Department of Pulmonary Immunology, The University of Texas Health Science Centre at Tyler, Tyler, TX, United States; ^6^ Cardiovascular Research Institute, University of California San Francisco, San Francisco, CA, United States; ^7^ Department of Medicine and Anaesthesia, University of California San Francisco, San Francisco, CA, United States

**Keywords:** COVID-19, D-dimer, fibrinolysis, fibrinogenolysis, comorbidity, meta-regression

## Abstract

**Background:**

Dynamic D-dimer level is a key biomarker for the severity and mortality of COVID-19 (coronavirus disease 2019). How aberrant fibrinolysis influences the clinical progression of COVID-19 presents a clinicopathological dilemma challenging intensivists.

**Methods:**

We performed meta-analysis and meta regression to analyze the associations of plasma D-dimer with 106 clinical variables to identify a panoramic view of the derangements of fibrinolysis in 14,862 patients of 42 studies. There were no limitations of age, gender, race, and country. Raw data of each group were extracted separately by two investigators. Individual data of case series, median and interquartile range, and ranges of median or mean were converted to SDM (standard deviation of mean).

**Findings:**

The weighted mean difference of D-dimer was 0.97 µg/mL (95% CI 0.65, 1.29) between mild and severe groups, as shown by meta-analysis. Publication bias was significant. Meta-regression identified 58 of 106 clinical variables were associated with plasma D-dimer levels. Of these, 11 readouts were negatively related to the level of plasma D-dimer. Further, age and gender were confounding factors. There were 22 variables independently correlated with the D-dimer level, including respiratory rate, dyspnea plasma K^+^, glucose, SpO2, BUN (blood urea nitrogen), bilirubin, ALT (alanine aminotransferase), AST (aspartate aminotransferase), systolic blood pressure, and CK (creatine kinase).

**Interpretation:**

These findings support elevated D-dimer as an independent predictor for both mortality and complications. The identified D-dimer-associated clinical variables draw a landscape integrating the aggregate effects of systemically suppressive and pulmonary hyperactive derangements of fibrinolysis, and the D-dimer-associated clinical biomarkers, and conceptually parameters could be combined for risk stratification, potentially for tracking thrombolytic therapy or alternative interventions.

## Introduction

The sustained COVID-19 pandemic has oversaturated the emergency and intensive critical care resources globally. Hypercoagulability has been evidenced in most critically ill patients by elevated D-dimer and fibrin degradation products (FDP), a decrease in platelet count, an incremental increase in the prothrombin time, and a rise in fibrinogen (1–9). Of these, patients with increased D-dimer are more vulnerable to worsen clinical consequences of COVID-19, with more severe complications, including requirements of ICU support (1–9).

Thromboembolism of COVID-19 patients is the fatal sequelae of hypercoagulation and fibrinolytic abnormalities. Pulmonary embolism (PE) and deep vein thrombosis (DVT) can cause respiratory failure in severely ill patients with COVID-19 (10–14). Postmortem pathology shows that small fibrinous thrombi in small pulmonary arterioles are very common. Activation of the coagulation cascade is further supported by endothelial tumefaction, pulmonary megakaryocytes in the capillaries, and endotheliitis (15–19). Elevated D-dimer is an indicator of the activation of the fibrinolysis system and removal of clots or extravascular collections of fibrin by plasmin. Compared with the consistent coagulopathy, however, the clinical ramifications of deranged fibrinolysis are not well studied and reviewed systematically.

Increased D-dimer level has not consistently been observed by all COVID-19 clinical studies, although it is a broadly applied biomarker for prognosis and outcomes of anti-thrombosis (20). The current explanations for the elevated D-dimer in critically ill patients are multiple, including “suppression of fibrinolysis”, “secondarily hyperactive fibrinolysis”, “consumption of fibrinolysis”, “fibrinolysis resistance”, and “fibrinolysis shutdown” (21, 22). To restore the coagulopathic changes of COVID-19, two diametrically different therapeutic regimes are in practice: fibrinolytic (alteplase-tPA) (10, 11, 23–28) and antifibrinolytic therapies (nafamostat and tranexamic acid (TXA)) (29–31). It is therefore imperative to clarify the role of pathophysiologic derangements of fibrinolysis in clinical outcomes that occur in COVID-19 patients. We, therefore, performed both meta-analyses and meta-regressions to explore the relationships between the plasma D-dimer level on admission with demographics, laboratory tests, fatal cardiopulmonary function, radiology, interventions, complications, and outcomes.

## Materials and Methods

**Figure 1:**
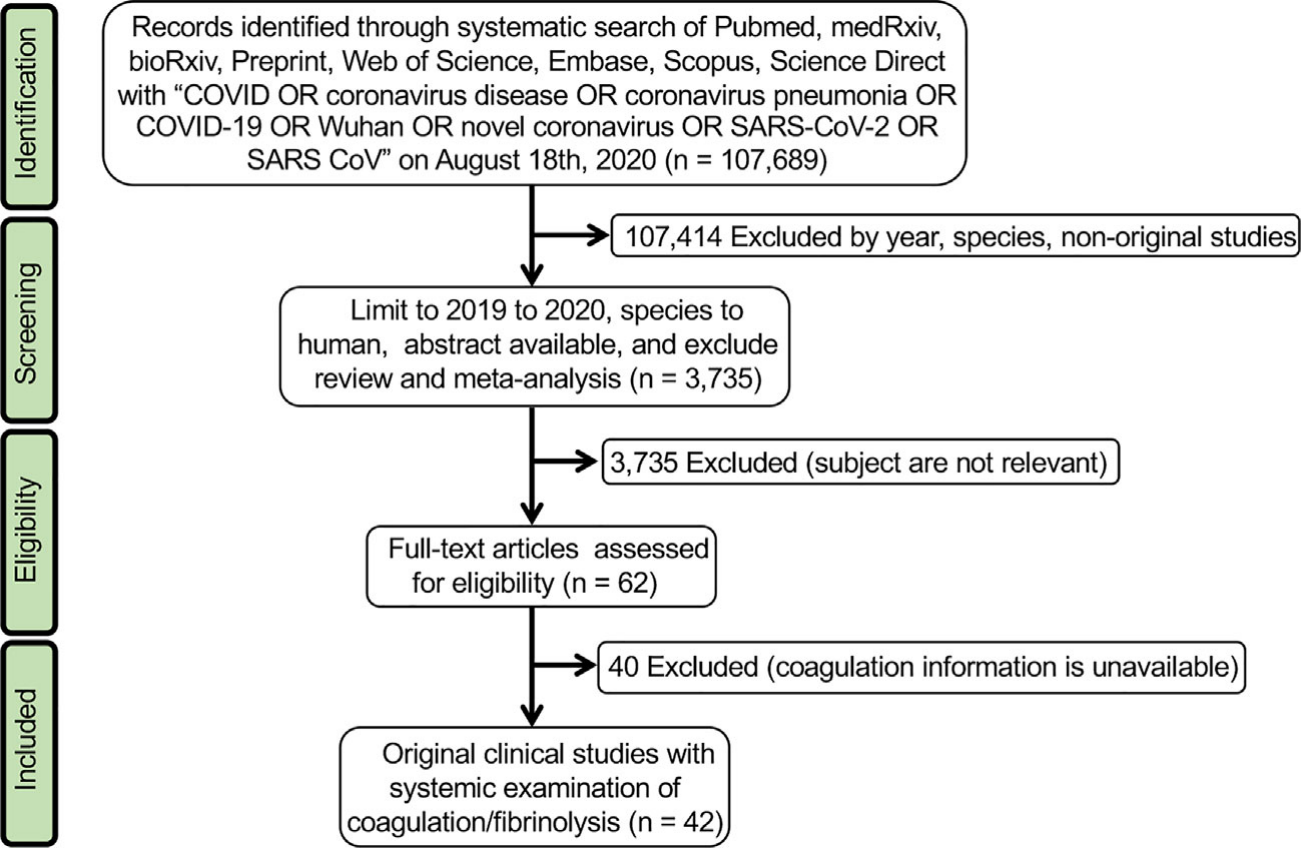
PRISMA 2009 flow diagram for searching included studies.

We conducted a systematic review of the literature in accordance with the methods recommended in the PRISMA guidelines (**Figure 1**).

### Literature Search

Two independent investigators searched the potential studies in the NCBI PubMed, EMBASE, Scopus, Web of Science, Google Scholar, and some preprint platforms, including the medRxiv, Preprint, and bioRxiv. The search strategy was (D-dimer OR fibrin OR proteolytic OR fibrinolysis OR coagulation OR thrombin OR platelet OR plasmin OR tPA OR fibrinolytic OR thrombolytic) AND (COVID-19 OR 2019-nCoV OR SARS-nCoV OR Wuhan OR SARS-CoV-2). Sorted COVID-19 and SARS-CoV-2 preprints were screened if available. The hits were limited to publication in the year 2019-2020. Studies published in some high-impact journals focusing on the fibrinolysis and coagulation systems were summarized (**Table 1**). Related articles published during the preparation of this manuscript were discussed.

**Table 1:** Demographic features of the analyzed 42 studies. *IQR. Mean ± S.D. # normal and D-dimer elevated group. (-) range from minimum to maximum.

Study	Journal	Region	Cases	Age (year)	Race	Male/%	D-dimer (µg/mL)	Mortality/%
Akalin E et al. (9)	NEJM	USA	36	60 (32 - 77)	AF/CC/MR	26/72	1.02 (0.32 - 5.19)	10/28
Borba MG et al. (32)	JAMA Network	Brazil	81	51.1 ± 13.9	CC/AA/MR	61/75.3	N/A	22/27.2
Chen G et al. (33)	JCI	China	21	56 (50, 65)*	Asian	17/81	0.5 (0.4, 1.8)*	4/19
Chen N et al. (34)	Lancet	China	99	55.5 ± 13.1	Asian	67/68	0.9 (0.5, 2.8)*	11/11
Chen T et al. (35)	BMJ	China	274	62 (44, 70)*	Asian	171/62	1.1 (0.5, 3.2)*	113/41.2
Cui S et al. (36)	J Thromb Haemost	China	81	59.9 ± 14.1	Asian	37/46	3 (0 - 8.2)	8/10
Du R et al. (37)	ERJ	China	179	56.7 ± 13.7	Asian	97/54.2	0.5 (0.3, 1.7)*	21/11.73
Du Y et al. (38)	AJRCCM	China	85	65.8 ± 14.2	Asian	62/72.9	5.16 ± 4.68	81/95.29
Feng Y et al. (39)	AJRCCM	China	476	53 (40, 64)*	Asian	271/56.9	0.58 (0.35, 1.48)*	38/8
Fogarty H et al. (40)	BJH	Ireland	83	62 ± 16.3	CC/AS/AF	55/66.27	0.88 (0.74, 3.46)*	13/15.7
Han H et al. (41)	CCLM	China	94	N/A	Asian	48/51	10.36 ± 25.31	N/A
Helm J et al. (13)	ICM	France	150	63 (53,71)*	CC/AA/MR	122/81.3	2.27 (1.16, 20.0)*	13/8.7
Huang C et al. (42)	Lancet	China	41	49 (41, 58)*	Asian	30/73	0.5 (0.3, 1.3)*	6/15
Lu JT et al. (8)	Lancet Infect Dis	China	577	55 (39,66)*	Asian	254/44	0.3 (0.1, 0.7)*	39/6.8
Mo P et al. (43)	CID	China	155	54 (42, 66)*	Asian	86/55.5	0.19 (0.12, 0.36)*	22/14.2
Oxley TJ et al. (44)	NEJM	USA	5	40.4 (33 - 49)	CC	4/80	3.66 (0.05 - 13.8)	0
Panigada M et al. (45)	J Thromb Haemost	Italy	24	56 (23 - 71)	CC	N/A	4.88 (1.2 - 16.95)	N/A
Paranjpe I et al. (46)	medRxiv	USA	2,199	65 (54 - 76)*	CC/AA/AS/MR	1293/58.8	1.31 (0.74, 2.44)*	310/14.10
Qiu H et al. (47)	Lancet Infect Dis	China	36	8.3 ± 3.5	Asian	23/64	0.29 ± 0.2	0
Ranucci M et al. (48)	J Thromb Haemost	Italy	16	61 (55, 65)*	CC	15/93.75	2.5 (1.6, 2.8)*	7/43.7
Rentsch CH et al. (49)	medRxiv	USA	585	66.1 (60.4,71.0)*	CC/AF/MR	558/95.4	N/A	17/2.9
Richard S et al. (7)	JAMA	USA	5,700	63 (52, 75)*	AF/AS/CC/MR	3437/60.3	0.44 (0.26, 0.87)*	553/21
Spiezia L et al. (50)	Thromb Haemost	Italy	22	67 ± 8	CC	20/90.91	5.34 ± 2.10	1/4.55
Tang N et al. (51)	J Thromb Haemost	China	449	65.1 ± 12.0	Asian	268/59.69	1.94 (0.9, 9.44)*	134/29.8
Wang D et al. (6)	JAMA	China	138	56 (22 - 92)	Asian	75/54.3	0.20 (0.12 - 0.40)	6/4.3
Wang L et al. (52)	J Infect	China	339	69 (65 - 76)*	Asian	166/49	1.20 (0.62, 3.25)*	65/19.2
Wang Y et al. (53)	AJRCCM	China	344	64 (52, 72)*	Asian	179/52.0	1.3 (0.5, 5.0)*	133/38.7
Wang YM et al. (5)	Lancet	China	158	66.0 (57, 73)*	Asian	89/56	N/A	22/15
Wang Z et al. (54)	CID	China	69	42 (35, 62)*	Asian	32/46	N/A	5/7.5
Wu C et al. (55)	JAMA Inter Med	China	201	51 (43, 60)*	Asian	128/63.7	0.61 (0.35, 1.28)*	44/21.9
Wu J et al. (56)	CID	China	80	46.1 ± 15.42	Asian	39/48.75	0.9 (0.4, 2.4)*	0
Xie J et al. (3)	Lancet	China	299	62 (50.8, 71)*	Asian	239/53.8	N/A	224/50.5
Xu X et al. (4)	BMJ	China	62	41 (32, 52)*	Asian	36/58	0.2 (0.2, 0.5)*	0
Yang F et al. (57)	I Med Virol	China	52	63 (34 - 98)*	Asian	28 (53.8)	1.7 (0.7, 3.3)*	11/21.2
Yang WJ et al. (58)	J Infect	China	149	45.11 ± 13.35	Asian	81/54.4	0.22 ± 0.28	0
Yang X et al. (59)	Lancet Respir Dis	China	52	59.7 ± 13.3	Asian	35/67	N/A	32/61.54
Yao Y et al. (60)	J Intensive Care	China	63#	63.0 ± 13.4	Asian	135/54.4	0.35 (0.23, 0.42)*	17/9.2
			185#				1.69 (0.91, 5.06)*	
Zhang JJ et al. (61)	Allergy	China	140	57 (25, 87)*	Asian	71/50.7	0.2 (0.1, 0.5)*	N/A
Zhang L et al. (62)	J Thromb Haemost	China	343	62 (48, 69)*	Asian	169/49.27	0.54 (0.20, 1.41)*	13/3.8
Zhang Y et al. (2)	NEJM	China	3	68 (65 - 70)	Asian	2/66.67	9.02 ± 10.37	N/A
Zhou F et al. (1)	Lancet	China	191	56 (46, 67)*	Asian	119/62	0.8 (0.4, 3.2)*	54/28.3
Zou Y et al. (63)	BioSci Trend	China	303	51 (16 - 88)	Asian	158/52.15	0.45 (0.31, 0.81)*	N/A

### Criteria for Study Selection and Data Extraction

All eligible studies meeting the following criteria were included: 1) the species was human, 2) the publications were original clinical investigations, and 3) the results were presented as or could be converted or digitized to mean ± SD (SDM) or percentage. Studies were excluded if they were: 1) reviews or editorial, single case reports, commentaries, or preclinical studies; 2) results that could not be converted or digitized to SDM or percentage; and 3) full articles or clinical data that were not available. Raw data of each sub-group at admission were extracted by ZLS and RZZ. Individual data of case series (2, 44), median and interquartile range (1, 4–8, 13, 33, 35, 39, 42, 43, 46, 48, 49, 52–54, 56, 57, 61, 62), and ranges of median or mean (9, 45, 63) were converted to SDM as described previously (64, 65). Percentages and SDMs were extracted directly from studies if available.

### Meta-Analysis

To perform meta-analysis with the STATA v.16.1, the studies with two groups or more were pooled to compute weighted mean differences (WMD) and 95% confidence intervals (95% CI) (64). Different units and methodologies were converted to unified ones; for example, ng/ml, mcg/ml, µ;g/L for D-dimer were computed to µ;g/mL for all studies. The stages of COVID-19 were defined by the original studies mostly based on the WHO Interim Guidance. Publication bias between selected studies was assessed with both the Egger’s and Begg’s tests using the metabias program. The stability of the results was confirmed by the random-effects trim and fill analyses using the metatrim program.

### Meta-Regression Analysis

The associations between D-dimer and demographic features, comorbidities, laboratory tests, radiographic results, treatments, hospitalization, outcomes, and complications were analyzed. D-dimer was considered as a covariate of other clinical variables. The standard errors of D-dimer were used to indicate the within-study variability, and the random-effect ReML method was applied. If observations were lesser than six, the results of this parameter were removed. All defined complications/diagnoses, i.e., ARDS, DIC, sepsis, were originally reported by the included studies.

## Results

### General Information

Following the PRISMA guideline, we included 42 key studies for meta-analysis and meta-regression (**Figure 1**). The demographic features, D-dimer, ARDS, and mortality were summarized in **Table 1**. In total, there were 14,862 laboratory-confirmed patients: 31 studies from China (5,961cases), 5 from the USA (8,525 cases), 3 from Italy (62 cases), and 1 from Ireland (83 cases), France (150 cases), and Brazil (81 cases), respectively. The age was from 1 to 98-year-old. The races included Asian, African, Caucasian, and mixed with an incidence of ARDS ranged from 0 to 100%, and a fatality ranged from 0 to 95.3%. D-dimer level ranged from 0 to 35.7 µ;g/mL, and 9 studies reported a normal value (< 0.5 µ;g/mL).

### Significant Publication Bias Caused by Divergent Study Designs

**Figure 2:**
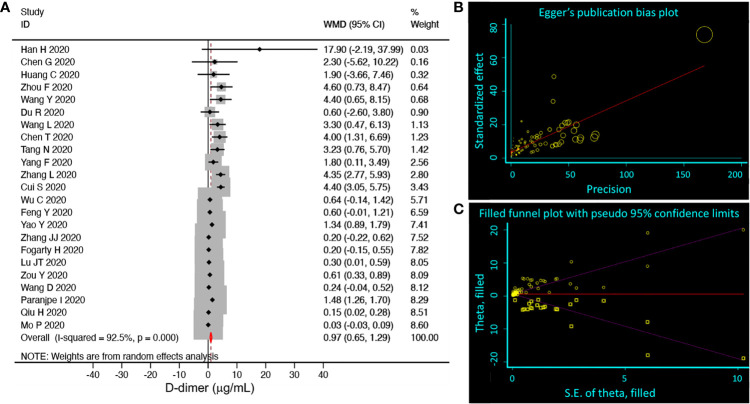
Random-effects meta-analysis of D-dimer level. **(A)** Forest plot. We selected studies that had subgroups, which could be divided into mild (including normal) and severe groups. We pooled weighted mean differences (WMD, black diamond, and gray square) and 95% CI (horizontal lines through the diamonds) of D-dimer from eligible 23 studies. Studies with only one group or moderate group were excluded. The red diamond represents the overall WMD. **(B)** Egger’s publication bias plot. N=89, P<0.001. **(C)** Filled funnel plot. P<0.001. Circle, raw data; square, pseudo data needed for symmetric distribution.

COVID-19, as a newly emerging infectious disease, the included clinical studies had divergent designs. We compared the D-dimer level between mild and severe groups of 23 studies (**Figure 2A**). Only a small increase of D-dimer (normal range <0.5 µ;g/mL), 0.97 µ;g/mL (95% CI 0.65, 1.29) was observed in the relatively severe group with significant publication bias (92.5%), which was corroborated by both the Begg’s (P=0.009) and Egger’s tests (P<0.001) (**Figure 2B**) and the random-effects filled funnel plot (**Figure 2C**, P<0.001). Similarly, significant variations caused by retrospective studies and case series were observed for both age (**Figure S1**) and mortality (**Figure S2**). Thus, it was considered to be inappropriate to perform meta-analysis without well-designed RCT (randomized controlled trials) studies. Instead, we hypothesized that D-dimer could serve as a critical covariate for clinical features.

### D-Dimer Correlated to Demographic Features

We performed meta-regression to detect the potential associations of D-dimer with 36 demographic characteristics of COVID-19 patients (**Table S2**). Of these, preexisting medical conditions, including any comorbidity, hypertension, diabetes, chronic lung diseases, and cerebrovascular diseases, were positively associated with D-dimer (P<0.05). Age, gender, blood pressure, and dyspnea/tachypnea positively correlated with D-dimer (P<0.05, **Table 2**). Moreover, the percentage of female and diastolic pressure was negatively correlated to D-dimer. Subgroup analysis showed a cutoff age was <65 years in the studies with two groups (<65 or ≥65), and further <50 in the studies with four groups (<50, ≥50, <60, ≥60, and ≥70) for a negative coefficient value (P<0.05, **Table S3**).

**Table 2:** D-dimer associated clinical variables (58 of 106 in total with a sample size ≥ 5, P < 0.05) identified by univariate-regression.

Characteristics	Univariate coeff	Obs	AR^2^	P value	Manifestations	Normal range†
Age (yr)	0.043 (0.022, 0.063)	80	24.06	< 0.001§	<50	NA
Male (%)	0.047 (0.024, 0.071)	78	22.79	< 0.001*	NA	NA
Female (%)	-0.047 (-0.071, -0.024)	78	22.79	< 0.001*	NA	NA
**Preexisting conditions**						
Comorbidity (%)	0.020 (0.002, 0.038)	45	14.57	0.030§		NA
Hypertension (%)	0.027 (0.010, 0.043)	60	28.01	0.002§	Comorbidity	NA
Diabetes (%)	0.042 (0.018, 0.066)	61	36.42	0.001§	Comorbidity	NA
Chronic lung diseases (%)	0.104 (0.045, 0.163)	62	20.92	0.001	Comorbidity	NA
Onset to admission (day)	0.179 (0.048, 0.310)	31	31.89	0.009§	Severity	NA
**Inflammatory and tissue injury markers**						
Platelet (× 10^9^/L)	0.014 (0.004, 0.024)	64	18.65	0.009*	Thrombocytopenia	125 - 350
WBC (× 10^9^/L)	0.518 (0.387, 0.649)	71	65.26	< 0.001	Leukocytosis	4 - 11
Neutrophil (× 10^9^/L)	0.447 (0.307, 0.586)	53	68.98	< 0.001*§	Neutrophilia	1.5 - 8
Lymphocyte (× 10^9^/L)	-0.606 (-1.115, -0.096)	70	7.70	0.020§	Lymphopenia	1.0 - 4.8
CD4^+^ T cell (/µl)	-0.004 (-0.008, -0.001)	21	29.00	0.018§	Inflammation	500 - 1,200
CD8^+^ T cell (/µl)	-0.008 (-0.012, -0.005)	21	75.33	< 0.001§	Inflammation	200 - 800
IL2R (U/mL)	0.003 (0.000, 0.005)	9	45.90	0.044	Cytokine receptor	100 - 500
IL6 (pg/mL)	0.011(0.001, 0.022)	28	-10.23	0.030	Pro-cytokine	≤ 1.8
IL8 (pg/mL)	0.122 (0.017, 0.227)	9	45.39	0.029§	Chemokine	< 57.8
TNFαpg/mL	0.543 (0.002, 1.085)	9	9.65	0.049§	Cytokine	≤ 2.8
Globulin (g/L)	0.167 (0.044, 0.291)	8	83.51	0.016*§	Hypoproteinemia	20 - 35
Fibrinogen (g/L)	1.077 (0.272, 1.882)	27	45.94	0.011*	Hyperfibrinogenemia	2 - 4
Ferritin (µ;g/L)	0.002 (0.001, 0.003)	23	13.05	0.008*§	Iron transport	30 - 400
CRP (mg/L)	0.013 (0.006, 0.021)	45	48.18	0.001*§	Inflammation	< 8.0
Albumin (g/L)	-0.158 (-0.249, -0.066)	41	29.29	0.001§	Hypoalbuminemia	35 - 55
PCT (ng/mL)	1.137 (0.720, 1.554)	53	50.76	< 0.001*§	Bacterial sepsis	< 0.5
ESR (mm/h)	0.009 (0.001, 0.017)	17	42.93	0.032	Inflammation	1 - 20
LDH (U/L)	0.007 (0.005, 0.009)	51	62.65	< 0.001*§	Tissue damage	140 - 280
Serum K^+^ (mmol/L)	2.187 (1.064, 3.309)	16	64.07	0.001*§	Hyperkalemia	3.6 - 5.2
Glucose (mmol/L)	0.978 (0.395, 1.562)	14	61.42	0.003*§	Hyperglycemia	4.4 - 7.2
Hemoglobin (g/L)	-0.157 (-0.246, -0.068)	37	27.70	0.001§	Anemia	121 - 172
Time for PCR-negative (day)	0.035 (0.021, 0.049)	8	100	0.001§	RNAaemia	NA
**Acute lung injury markers**						
Respiratory rate (/min)	0.749 (0.269, 1.229)	19	-1.43	0.004*§	Lung function	12 - 20
Dyspnea/tachypnea (%)	0.019 (0.007, 0.030)	45	23.35	0.002*§	Lung injury	NA
SpO2 (%)	-0.183 (-0.303, -0.063)	10	46.77	0.008*§	Hypoxia	95 - 100
Ventilation (%)	0.019 (0.010, 0.028)	71	28.50	< 0.001*§	Lung injury	NA
CURB 65 (lung)	1.945 (1.011, 2.879)	8	87.65	0.002	Lung injury	NA
Onset to dyspnea (day)	0.090 (0.021, 0.158)	11	69.35	0.016*	Lung injury	NA
**Acute kidney injury markers**						
eGFR (ml/min/1.73m^2^)	-0.045 (-0.089, -0.001)	9	37.84	0.047§	Kidney injury	90
BUN (mmol/L)	0.651 (0.493, 0.810)	31	87.22	< 0.001*§	Kidney injury	3 - 7
**Acute liver injury biomarkers**						
Total bilirubin (µ;mol/L)	0.245 (0.138, 0.353)	36	50.40	< 0.001*§	Liver injury	5.1 - 17
Alkaline phosphatase (U/L)	0.083 (0.042, 0.125)	5	100.00	0.008*	Liver diseases	20 - 140
γ-glutamyl transpeptidase (GGT) (U/L)	0.193 (0.063, 0.324)	8	73.76	0.011	Liver diseases	9 - 48
ALT (U/L)	0.072 (0.048, 0.097)	62	60.11	< 0.001*§	Liver injury	19 - 33
AST (U/L)	0.052 (0.035, 0.070)	53	56.96	< 0.001*§	Liver injury	7 - 56
**Cardiac/skeletal muscle injury markers**						
Systolic pressure (mmHg)	0.018 (0.007, 0.029)	21	46.67	0.004*§	Cardiovascular	90 - 120
Diastolic pressure (mmHg)	-0.155 (-0.271, -0.039)	6	89.60	0.021	Cardiovascular	60 - 80
Myoglobin (ng/mL)	0.012 (0.004, 0.019)	11	83.51	0.006§	Rhabdomyolysis	0 - 85
CK (U/L)	0.006 (0.003, 0.009)	51	47.26	< 0.001*§	Muscle injury	22 - 198
CK-MB (U/L)	-0.032 (-0.057, -0.007)	24	45.47	0.014§	Myocardial infarction	5 - 25
**Complications**						
ARDS (%)	0.033 (0.023, 0.043)	48	56.63	< 0.001*§	Lung injury	NA
Secondary infection (%)	0.018 (0.012, 0.025)	17	85.62	< 0.001§	Systemic	NA
Sepsis (%)	0.029 (0.015, 0.043)	34	36.52	< 0.001*§	Systemic	NA
Acute cardiac injury (%)	0.048 (0.032, 0.065)	42	49.65	< 0.001*§	Heart injury	NA
Thrombosis	0.041 (0.011, 0.071)	17	38.05	0.011	Systemic	NA
Acute kidney injury (%)	0.029 (0.007, 0.052)	28	-1.61	0.014§	Kidney injury	NA
DIC (%)	0.042 (0.001, 0.083)	6	95.63	0.048	Systemic	NA
Acute liver injury (%)	0.055 (0.012, 0.098)	19	38.29	0.015§	Liver injury	NA
**Prognosis**						
Mortality (%)	0.031 (0.022, 0.040)	72	52.65	< 0.001*§	Death	NA
Discharged (%)	-0.007 (-0.012, -0.002)	45	11.96	0.010§	Survival	NA

CI, confidence interval; Obs, the number of groups from the included studies; WBC, white blood cells; CK, creatine kinase; CK-MB, creatine kinase myocardial band; ALT, alanine aminotransferase; AST, aspartate aminotransferase; BUN, blood urine nitrogen; eGFR, estimated glomerular filtration rate; LDH, lactate dehydrogenase; PCT, Procalcitonin; CRP, C-reactive protein; ESR, erythrocyte sedimentation rate; IL2R, interleukin 2 receptor; TNFα, tissue necrosis factor α; CURB 65, CURB-65 Severity Score; ARDS, acute respiratory distress syndrome; TE, thromboembolism; DIC, disseminated intravascular coagulation. **†** include the range of both male and female. P < 0.05 after removing the confounding variable age (*) or male (§) using bivariate regression.

### D-Dimer Is a Correlate of Laboratory Tests but Not Radiologic Readouts

To analyze D-dimer’s correlation with 62 laboratory tests and radiological readouts, meta-regression was conducted for individual variables and summarized in **Table S4**. There were 32 laboratory tests significantly associated with D-dimer (**Table S4**). In addition, FDP tended to associate with D-dimer levels (P=0.058, N=10). These tests could be roughly grouped as 1) inflammation and tissue injury markers, 2) acute lung injury markers, 3) acute kidney injury markers, 4) acute liver injury markers, and 5) cardiac/skeletal muscle injury markers (**Table 2**).

### D-Dimer Is Associated With Mechanical Ventilation

**Figure 3:**
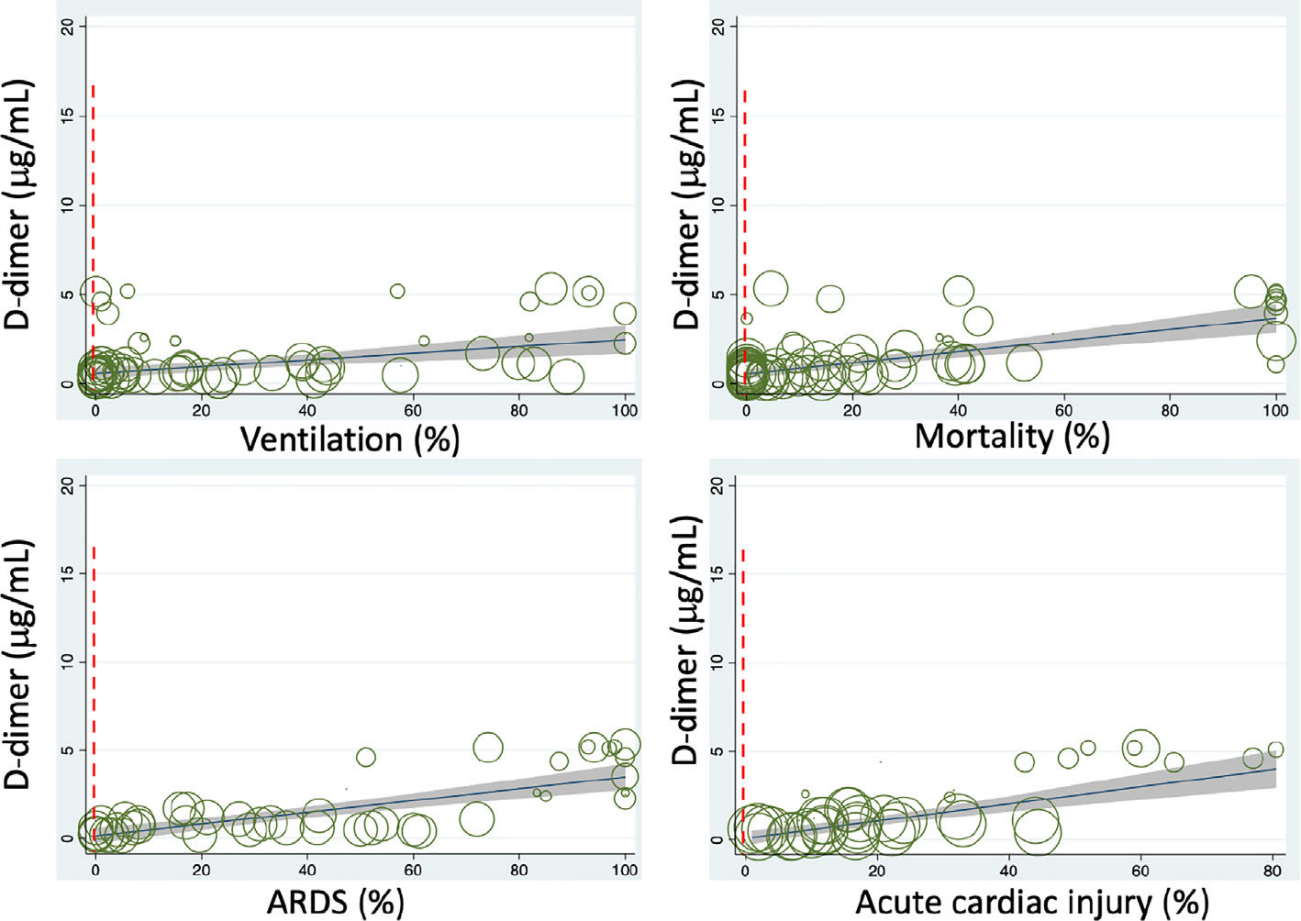
Meta-regressions of plasma D-dimer level on admission with some clinical variables. Dashed vertical red lines indicate the normal range.

To evaluate the relationship between interventions and D-dimer, we associated 11 therapies with the D-dimer level (**Tables S5** and **2**). Interestingly, mechanical ventilation was positively associated with D-dimer (N=71, P<0.001, **Figure 3**). Immune enhancement therapy (P=0.084, N=24) was also found to correlate with D-dimer if additional observations were available to increase the sample size.

### D-Dimer Serves as an Independent Risk Factor for Fatal Organ Injury and Systemic Conditions

To examine if D-dimer is an independent risk factor for deadly complications, we analyzed the dependence of fatal organ injury and systemic disorders using the metareg program. The results were summarized in **Tables S6** and **2**. Acute lung (ALI/ARDS), heart, kidney, and liver injuries were significantly associated with D-dimer (P<0.05, **Figure 3**). In addition, four systemic complications, i.e., sepsis, secondary infection, disseminated intravascular coagulation (DIC), and coagulopathy, showed significant associations with D-dimer. Acute brain injury and acidosis showed a tendency to associate with the D-dimer. Together, both acute fatal organ injury and systemic complications could be predicted by D-dimer.

### D-Dimer Predicts Severity, Hospitalization, and Outcomes

Given the correlation of D-dimer with the demographic features, abnormal laboratory tests, interventions, and severe complications, we hypothesized that D-dimer is an independent indicator for these disease progression (CURB 65 score, onset to admission, onset to dyspnea), hospitalization (discharged, time is taken to turn SARS-CoV-2 PCR negative), and mortality. We analyzed the correlation of D-dimer and these clinical readouts and summarized in **Tables S7** and **2**. D-dimer was positively associated with the severity of lung injury (CURB 65), the days from the onset to admission, onset to dyspnea, time is taken to be PCR negative, and overall mortality (**Figure 3**). In contrast, the discharge rate was negatively related to D-dimer, demonstrating D-dimer’s capability to serve as a prognostic variate for outcomes.

### Exclusion of Age and Gender as Confounding Factors

**Figure 4:**
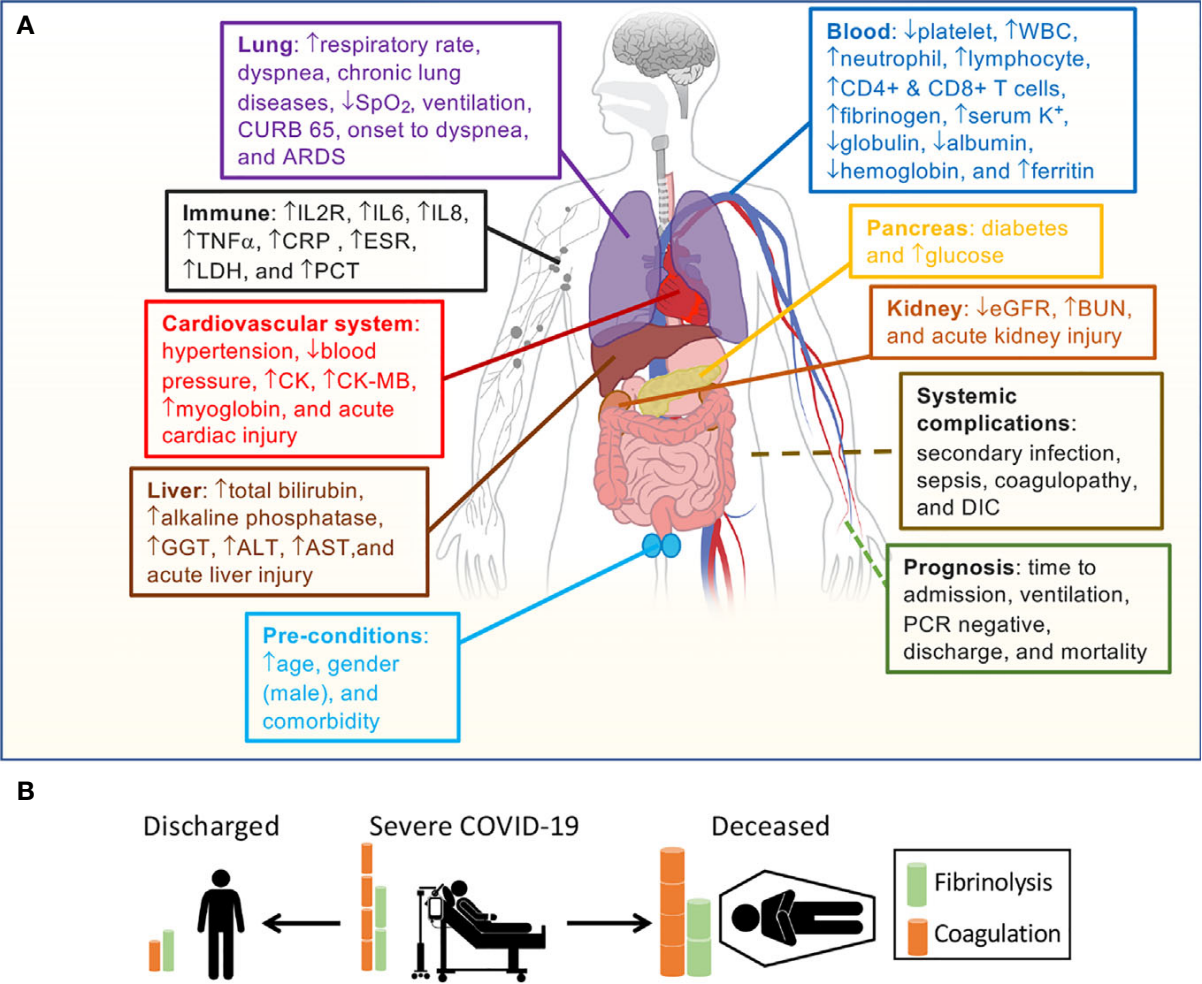
Correlation of D-dimer elevations and aberrant fibrin deposition with organ dysfunction. **(A)** D-dimer-associated clinical parameters sorted by the system and clinical relevance. **(B)** Outcomes correlate with D-dimer levels based on our hypothesis.

Age and gender have been identified as preexisting medical conditions associated with COVID-19 resulting in higher mortality (3, 35, 62). We eliminated their effects on the association of D-dimer with 61 identified variables in bivariate meta-regression analyses. Age was a significant confounding factor of 27 variables, and 26 variables were still associated with the D-dimer independent of age (**Table S8**). In contrast, 31 variables were disassociated with the D-dimer. By comparison with age, the male gender was a much weaker confounding covariate (**Table S9**). Males were significantly associated with 11 variables and results in the dissociation of 14 variables with D-dimer. The association of 28 and 42 variables with D-dimer was still significant after considering age and male as a covariate, respectively (**Table 2**). Age-affected 18 variables include comorbidity, hypertension, diabetes, lymphocyte, CD4^+^ and CD8^+^ T cells, IL8, TNFα, eGFR, hemoglobin, albumin, CK-MB, onset to admission, onset to PCR negative, discharged, secondary infection, acute kidney injury, and acute liver injury. There were 10 variables confounded by both age and male, including diastolic pressure, chronic lung diseases, WBC, IL2R, IL6, ESR, GGT, CURB 65 score, coagulopathy, and DIC. These four variables were markedly affected by covariate male: platelets, fibrinogen, alkaline phosphatase, and onset to dyspnea. Finally, 22 variables were significantly associated with D-dimer: respiratory rate, systolic pressure, dyspnea, serum K^+^, neutrophils, globulin, CRP, ferritin, LDH, PCT, SpO_2_, blood glucose, BUN, total bilirubin, ALT, AST, CK, mortality, ventilation, ARDS, sepsis, and acute cardiac injury (**Figure 4**).

## Discussion

We aimed to systematically analyze the relationships between circulating D-dimer level and clinical variables in critically ill COVID-19 patients. There is a range of plasma D-dimer levels on hospital admission. The directions of dynamically changed FDPs for hospitalized patients are different between discharged and deceased cohorts. Our meta-regression analysis revealed that plasma D-dimer is associated with comorbidities, demographics, some laboratory tests, radiology, hospitalization, complications, and outcomes. These results suggest that in addition to serving as an independent predictor for fatality, severity and could potentially serve as a marker for daily monitoring of thrombolytic therapy, D-dimer is a specific biomarker that interacts with other coagulation molecules, inflammatory cytokines, and markers for organ/tissue injury. Of note, the interplay of acute-phase proteins with fibrinogen and D-dimer suggests that infection-induced inflammation (cytokines and chemokines) initiates a state of hyperfibrinolysis. This notion is supported by D-dimer’s disassociation with the entire coagulation panel (PT, APTT, factor VIII and XI, TAT) (66, 67). In general, hyperfibrinolytic homeostasis maintains vascular patency and normal organ function under physiological conditions (**Figure 4B**). SARS-CoV-2 and co-bacterial infection initiate a hypercoagulable state followed by hyperfibrinolysis in COVID-19. If hyperfibrinolysis can counter excessive coagulopathy, then the patients could be protected against thrombosis. Otherwise, insufficient local hyperfibrinolysis in the lung of non-survivors will be exhausted.

Age is associated with an increased D-dimer level in COVID-19 patients at admission as a covariate or independent prognostic marker for the outcomes of COVID-19. The cutoff value of D-dimer (0.5 µ;g/mL) is age-dependent for healthy cohorts (68, 69). Our subgroup analysis is consistent with the concept that adults older than 50 approach the threshold of the D-dimer levels seen in normalcy (**Table S9**). The difference in D-dimer between men and women is minor in a healthy population (69). D-dimer’s positive association with the percentage of male patients in COVID studies suggests more severe cases in men than women when admitted.

**Figure 5:**
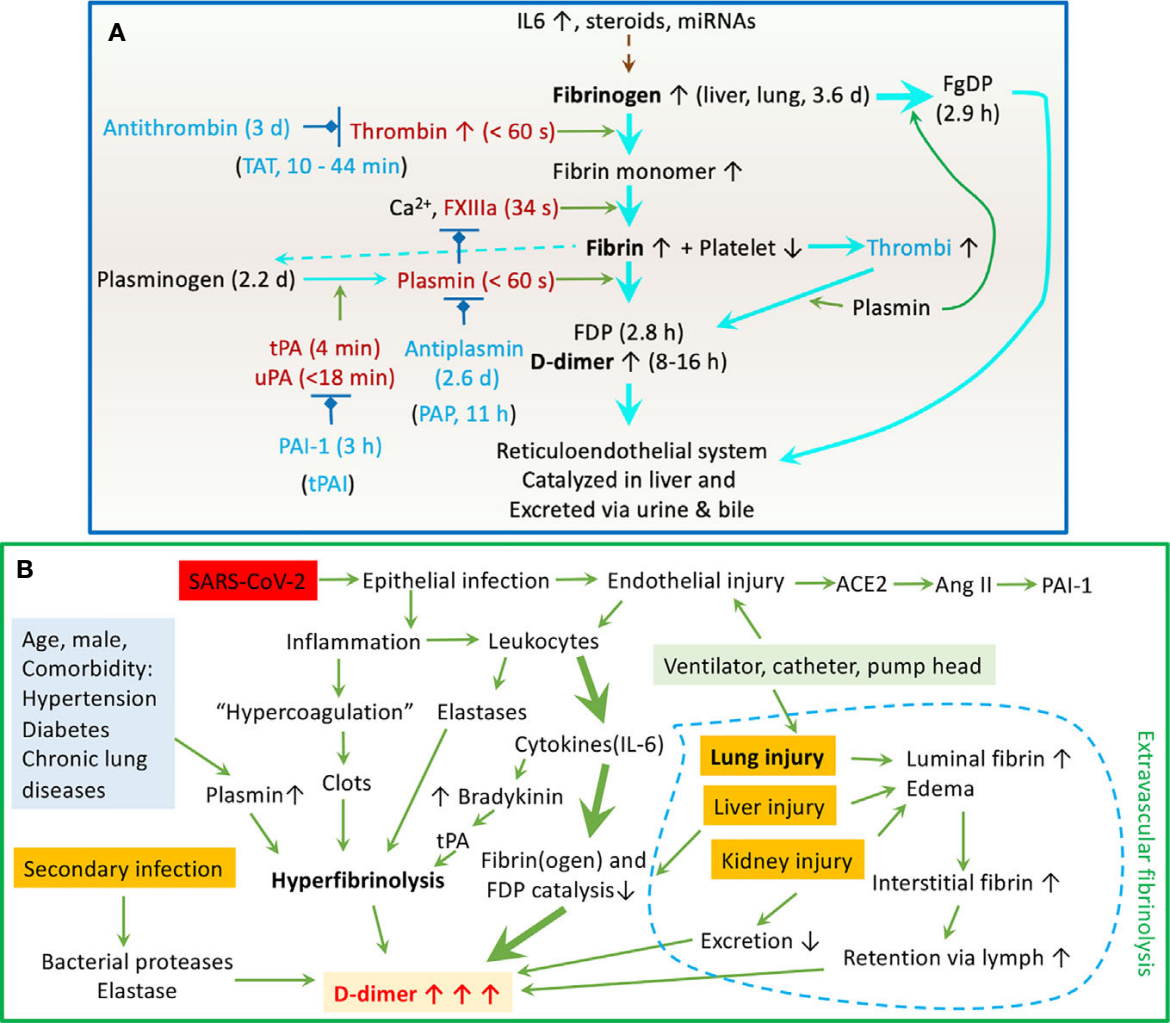
Schematic mechanisms for the dynamic D-dimer level in COVID-19 patients. **(A)** Regulation of the fibrinolysis system. The half lifetime for crucial components is given in followed brackets. **(B)** Clinicopathological mechanisms for elevated plasma D-dimer in COVID-19 patients.

Soluble fibrinogen is synthesized in the liver (1.7-5g/d) primarily and others, including the bone marrow, brain, lung, and gastrointestinal epithelium (**Figure 5A**). It mainly distributes in the plasma (75%), interstitial fluid (16%), platelets, and lymph (70, 71). IL6 and other proinflammatory cytokines/chemokines, steroids, and miRNAs upregulate the fibrinogen synthesis up to 10-fold during the acute phase of injury and infection. Fibrinogen (2-3%) can be turned over to fibrin monomers by thrombin and cleaved by plasmin, and the process termed fibrinogenolysis (70, 72). Fibrinogen degradation products (FgDP) are 2- or 3-fold that of plasma D-dimer but with a shorter half lifetime (2.8 h vs 16 h for D-dimer) (70, 72). Crosslinked fibrin is formed in the presence of FXIIIa. At the endothelial cell surface of injured blood vessels, fibrin(ogen) “glues” the plugs formed by the aggregation of platelets to develop thrombi (clots). Excessive fibrin deposition and inflammation activate endothelial cells to produce tPA and urokinase plasminogen activator (uPA) (70, 71). Either tPA or uPA is capable of cleaving hepatocyte-derived plasminogen to activate plasmin. Plasmin proteolytically cleaves fibrin within the thrombi into FDP and D-dimers, the end products of fibrinolysis. Given the very short half lifetime (in seconds or minutes) of endogenous thrombin, tPA, uPA, and plasmin, and the overwhelming antithrombin, plasminogen activator inhibitor 1 (PAI-1), and plasmin inhibitors with a much longer lifetime in the plasma (73), the primary cleavage of plasminogen and fibrin may predominately take place at the surface of clots. Eventually, FgDP and FDP (D-dimer) will be catabolized in the liver and captured by the reticuloendothelial system and excreted to the bile (70, 71). Another clearance pathway is *via* the kidney to excreted to urine (70, 71). The D-dimer assay has routinely been applied for excluding PE, deep venous thromboembolism, and DIC, as well as a marker for monitoring the effects of fibrinolytic/thrombolytic therapy.

Our regression analysis reveals that elevated D-dimer is associated with a broad spectrum of immune responses to SARS-CoV-2 infection, including increased pro-inflammatory cytokines (IL2R, IL6, IL8, and TNFα), acute phase proteins (CRP/C-reactive protein, fibrinogen, ferritin, and albumin), and inflammation indicators (ESR-erythrocyte sedimentation rate, PCT-procalcitonin, globulin, white blood cells, neutrophil, lymphocyte, and CD4^+^ & CD8^+^ T cells). Moreover, the days for reversion of the PCR test to negative is related to the D-dimer level. These correlations support the concept that interactions between high levels of circulating cytokines and hyperfibrinolysis may be functionally correlated. The binding of the spike proteins of SARS-CoV-2 to the ACE2 receptor in host respiratory epithelial cells downregulates the protective ACE2/Ang1-7/Mas axis, leading to increased expression of PAI-1 (74). Airway and lung epitheliitis releases pro-inflammatory cytokines to attract leukocytes from the blood. Moreover, infiltrated immune cells are activated and unleashed to attach normal lung tissues by releasing overwhelming cytokines. These cytokines upregulate positive acute-phase protein (i.e., fibrinogen), TF, and trypsin expression and inhibit negative proteins (i.e., albumin). Trypsin-activated matrix metalloproteinases break down the basolateral membrane and interstitial extracellular matrix. Further, endotheliopathy occurs in infected capillaries to initiate a local hypercoagulable state. The kinin-bradykinin is activated by IL6 to stimulate tPA expression in endothelial cells (74). Deposition of fibrin (clotting) activates endothelial cells to express more IL8, which suppresses clot lysis time (75). This, combined with the hypoxia-causing eryptosis, maybe the reason for IL8 associating with high mortality. ESR is associated with the severity of COVID-19 patients (76). In addition to carrying oxygen, erythrocyte-bound streptokinase and tPA break down the clots. Extrathyroidal produced PCT, if maintained at an elevated level by cytokines, is an indicator of poor outcomes of COVID-19 (77). It has been used as a marker for co-bacterial infection in septic shock and influenza. Neutrophil extracellular traps (NETs) may contribute to organ damage and promote thrombosis and fibrinolysis *via* elastase, so do lymphocytes/macrophages. Hepatocyte-synthesized globulins and albumin are involved in liver function, coagulation, and anti-inflammation. Their reduced levels by vast consumption predict a poor outcome. Albumin acts as an anticoagulant and antiplatelets and increases vascular permeability (78), which seems to explain the link between elevated D-dimer and hypoalbuminemia (**Figure 5B**).

The association of laboratory tests and complications can be explained by cell death. The lysis of platelets, erythrocytes, and other cells may contribute to higher serum K^+^ levels and reduced hemoglobin. This is supported by the association of D-dimer with LDH level and serum K^+^ and hemoglobin. At the organ level, D-dimer is associated with acute lung injury/ARDS, including hypoxia (respiratory rate, dyspnea, SpO_2_, CURB 65, and the onset of dyspnea). As a key early target organ of SARS-CoV-2 infection, both intra- and extravascular fibrin degradation in the alveolar sacs and interstitium could be the major site responsible for these associations. Hypoxia occurs in patients with the lungs with diffuse alveolar damage, a result in part of fibrin deposition and degradation. Moreover, mechanical ventilation with positive pressure could facilitate the retention of extravascular generated D-dimers. Associations of D-dimer with liver, kidney, and cardiac injury have been reported for other diseases (15, 17, 18, 79, 80). In addition, Pancreatic function and or metabolism of glucose may be dysfunctional, as shown by the association of D-dimer and blood glucose level. Our results demonstrate that D-dimer may serve as a biomarker to predict the damage of these organs by COVID-19 infection. Furthermore, the associations of D-dimer with systemic complications, including bacterial co-infection, septic shock, DIC, and thromboembolic events in large blood vessels, indicate high incidences and poor outcomes in critically ill patients.

Our results showed a strong positive association of elevated D-dimer on admission with mortality, indicating the prognostic value of an elevated D-dimer for the high risk of death. This is further corroborated by the positive correlation between D-dimer and days from onset to admission, the need for ventilation and the days are taken for PCR test reversion to negative. Another line of supportive evidence is the adverse relation of D-dimer and discharge probability. This data shows that prompt admission and clearance of the SARS-CoV-2 virus may alleviate the severity and reduce fatal events by preventing hyperfibrinolysis and inflammation. D-dimer, as a prognostic marker, is supported by other reviews (81–84) and clinical studies (1, 62). Patients with severe COVID-19 maybe those who were ill for a longer period than mild controls. This is supported by the association of the D-dimer level and the days from onset to admission (**Table 2**). Alternatively, the progression of the disease may be much quicker in critically ill patients at admission.

We have systematically reviewed the literature using meta-regressions and meta-analyses to define associations between elevated D-dimer and clinical multi-variants for the first time. Our study demonstrates: 1) D-dimer and other clinical variables’ associations indicate either a relationship that could be cause-effect or indirect. 2) Few D-dimer-associated variables have been confirmed or could be prognostic biomarkers for developing fatal events and in-hospital mortality. 3) Extremely elevated plasma D-dimer seems to be the consequence of hyperfibrinolysis predominately in the pulmonary capillaries and other organs. 4) A dynamic increase in the D-dimer level may be associated with thromboembolism and higher fatality, while we infer that a continuous decline by daily testing will generally lead to recovery.

Because COVID-19 is a newly emerging disease, most of the included studies are descriptive, case series, retrospective, single-center, and observational. Most of the studies included are from China/Asia, where the pandemic was discovered. Diversity in cohorts exists from children to seniors associated with variant comorbidities and complications. Inconsistent grouping strategies between studies pose a challenge for meta-analysis, for example, ICU vs non-ICU, ARDS vs non-ARDS, control vs COVID-19, survivor vs non-survivor, severe vs non-severe, VTE vs non-VTE, death vs recovered, mild vs moderate, moderate vs severe, normal vs abnormal D-dimer, etc. We cannot exclude the potential pre- and post-test deviations regarding the methodology for D-dimer assays. Meta-regression but not meta-analysis may partially mitigate these deviations. Also, a cutoff of D-dimer level could not be computed. Of note, D-Dimer level on admission could not be a biomarker associated with all clinical readouts.

We systematically analyzed the associations of D-dimer on admission with more than 100 clinical readouts. Our results demonstrate that elevated D-dimer could be inter-regulated by a spectrum of clinical variables, including preexisting conditions, inflammation, organ injury, abnormal glucose, complications, and outcomes. The clinical relevance of elevated D-dimer may be multifaceted.

## Author Contributions

H-LJ, ZS, and RZ were responsible for searching the literature, reviewing extracted data, meta-analyzing data, and preparing drafts of this manuscript. H-LJ, SI, AK, S-LL, MM, and GY improved the final manuscript, and all authors approved submission.

## Funding

This study was funded in part by the grants: HL87017 (HLJ), HL095435 (MAM), HL134828 (MAM), HL130402 (AK & SI), AI112381(SLL), AI150473 (SLL), HL154103 (SI & AK), and HL14285301 (SI). The funders had no role in study design, data ex- traction, data analysis, data interpretation, or writing of this study.

## Conflict of Interest

The authors declare that the research was conducted in the absence of any commercial or financial relationships that could be construed as a potential conflict of interest.
