# Supplementary material for: Associations of D-Dimer on Admission and Clinical Features of COVID-19 Patients: A Systematic Review, Meta-Analysis, and Meta-Regression
Source: Front Immunol. 2021 May 7;12:691249. doi: 10.3389/fimmu.2021.691249 (PMC8138429; doi:10.3389/fimmu.2021.691249)
Supplement: Supplementary file 1 [file Presentation_1.pdf]

Associations of D-dimer on admission and clinical features of COVID-19 patients: A systematic review and meta-analysis

Runzhen Zhao[1,*], Zhenlei Su[2,*], Andrey A. Komissarov[1,3], Shan-Lu Liu[4], Guohua Yi[5], Steven Idell[1,3], Michael A. Matthay[6,7], Hong-Long Ji[1,3]

[1]Department of Cellular and Molecular Biology, University of Texas Health Science Centre at Tyler, Tyler, TX, USA. [2]Department of Respiratory and Critical Care Medicine, Xinxiang Central Hospital, Xinxiang, Henan, China. [3]Texas Lung Injury Institute, The University of Texas Health Science Centre at Tyler, Tyler, TX, USA. [4]Department of Veterinary Biosciences, The Ohio State University, Columbus, OH, USA. [5]Department of Pulmonary Immunology, The University of Texas Health Science Centre at Tyler, Tyler, TX, USA. [6]Cardiovascular Research Institute, University of California San Francisco, San Francisco, CA, USA. [7]Department of Medicine and Anaesthesia, University of California San Francisco, San Francisco, CA, USA.

* These authors equally contributed to the study

Running title: Fibrin degradation of COVID-19

Correspondance

Hong-Long (James) Ji, james.ji@uthct.edu

Figure S1. Random-effects meta-analysis of age. **A**. Forest plot of 25 studies. See Figure 1 legend for details. **B**. Egger's publication bias plot. N=100, P<0.001. **C**. Filled funnel plot. P<0.001. Circle, raw data; square, pseudo data needed for symmetric distribution.

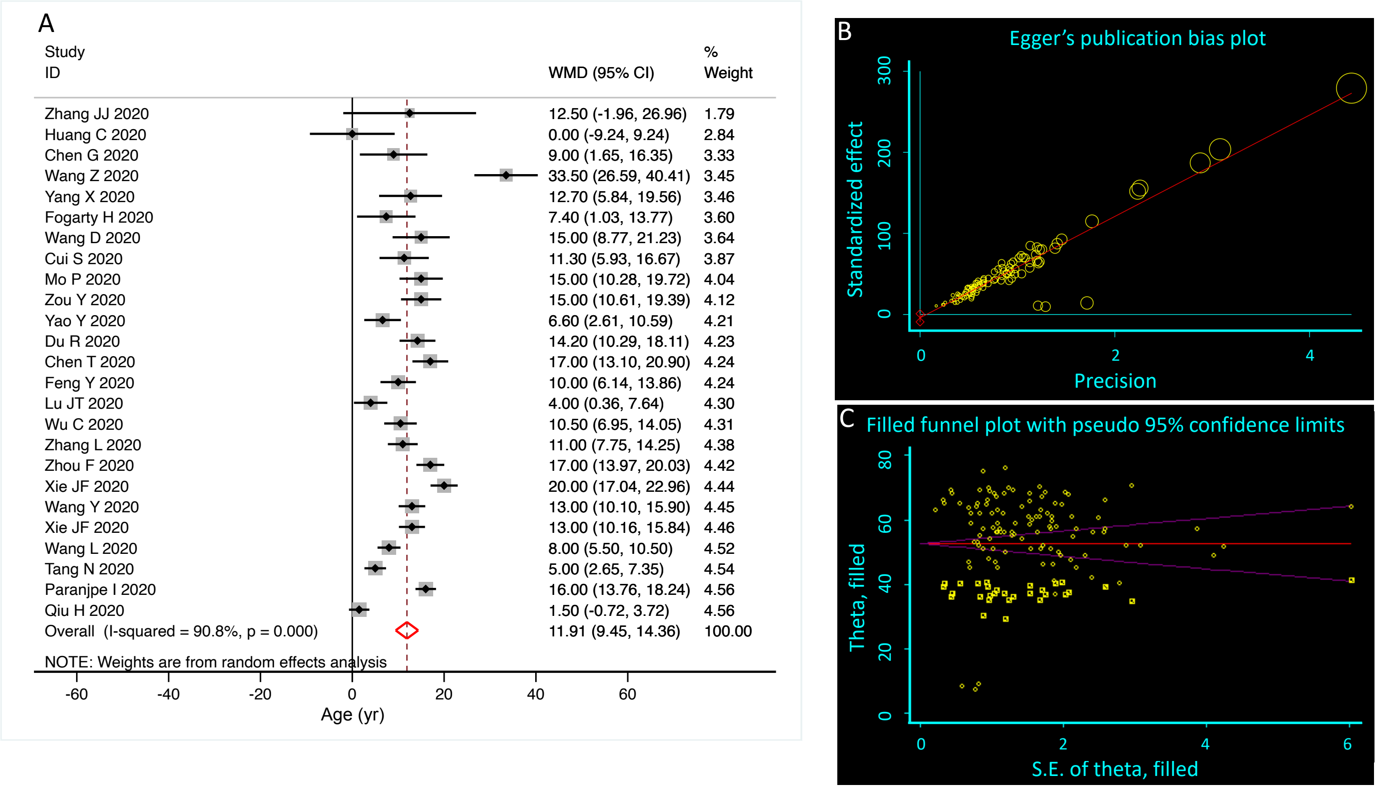

Figure S2. Random-effects meta-analysis of mortality (%).

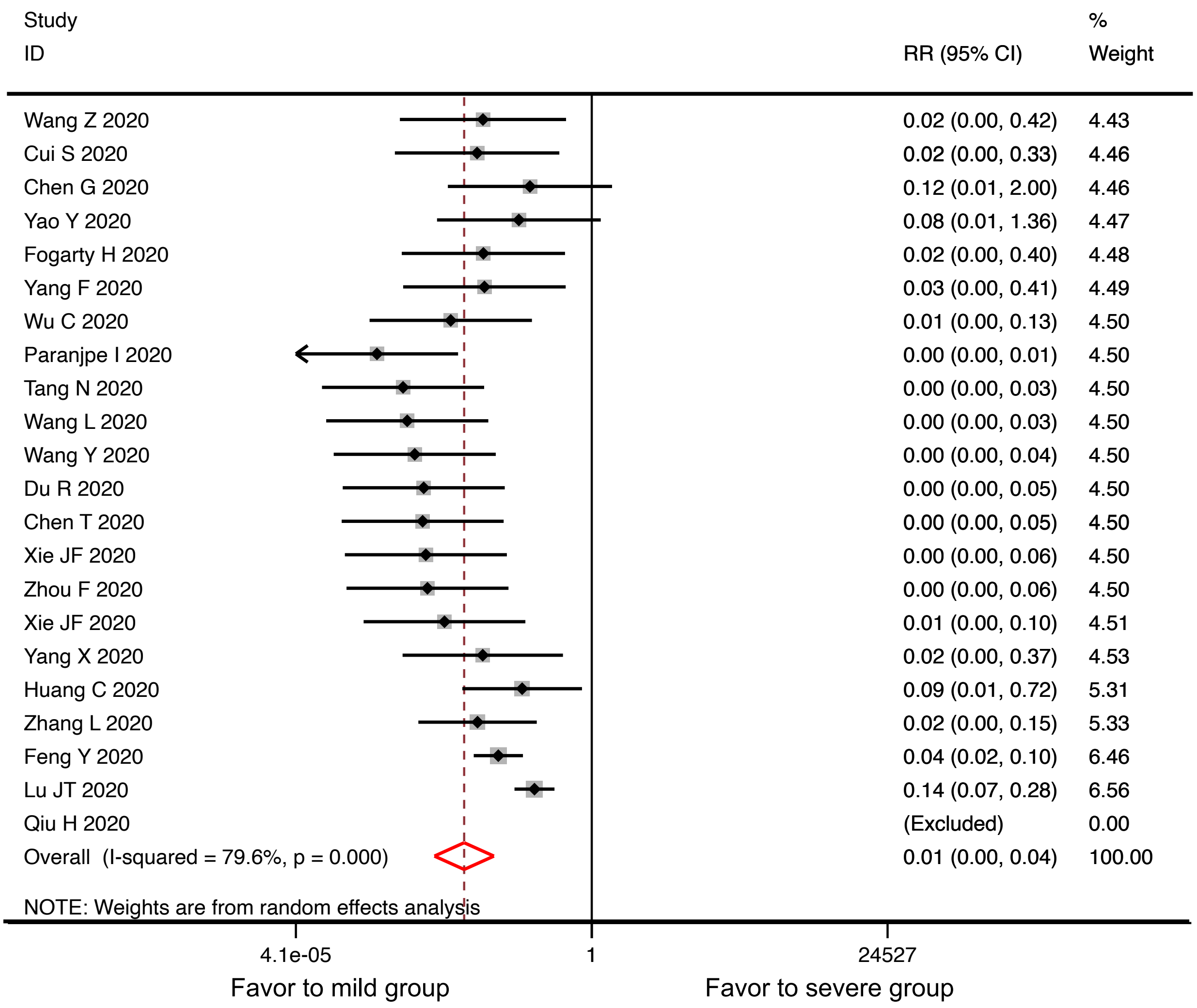

Table S1. PRISMA 2009 Checklist.

Section/topic	#	Checklist item	Reported on page #
**TITLE**			
Title	1	Identify the report as a systematic review, meta-analysis, or both.	1
**ABSTRACT**			
Structured summary	2	Provide a structured summary including, as applicable: background; objectives; data sources; study eligibility criteria, participants, and interventions; study appraisal and synthesis methods; results; limitations; conclusions and implications of key findings; systematic review registration number.	2
**INTRODUCTION**			
Rationale	3	Describe the rationale for the review in the context of what is already known.	3
Objectives	4	Provide an explicit statement of questions being addressed with reference to participants, interventions, comparisons, outcomes, and study design (PICOS).	3
**METHODS**			
Protocol and registration	5	Indicate if a review protocol exists, if and where it can be accessed (e.g., Web address), and, if available, provide registration information including registration number.	4
Eligibility criteria	6	Specify study characteristics (e.g., PICOS, length of follow-up) and report characteristics (e.g., years considered, language, publication status) used as criteria for eligibility, giving rationale.	4
Information sources	7	Describe all information sources (e.g., databases with dates of coverage, contact with study authors to identify additional studies) in the search and date last searched.	4
Search	8	Present full electronic search strategy for at least one database, including any limits used, such that it	4

		could be repeated.	
Study selection	9	State the process for selecting studies (i.e., screening, eligibility, included in systematic review, and, if applicable, included in the meta-analysis).	4
Data collection process	10	Describe method of data extraction from reports (e.g., piloted forms, independently, in duplicate) and any processes for obtaining and confirming data from investigators.	4
Data items	11	List and define all variables for which data were sought (e.g., PICOS, funding sources) and any assumptions and simplifications made.	4
Risk of bias in individual studies	12	Describe methods used for assessing risk of bias of individual studies (including specification of whether this was done at the study or outcome level), and how this information is to be used in any data synthesis.	4
Summary measures	13	State the principal summary measures (e.g., risk ratio, difference in means).	4
Synthesis of results	14	Describe the methods of handling data and combining results of studies, if done, including measures of consistency (e.g., I^2) for each meta-analysis.	4

Section/topic	#	Checklist item	Reported on page #
Risk of bias across studies	15	Specify any assessment of risk of bias that may affect the cumulative evidence (e.g., publication bias, selective reporting within studies).	4
Additional analyses	16	Describe methods of additional analyses (e.g., sensitivity or subgroup analyses, meta-regression), if done, indicating which were pre-specified.	4

**RESULTS**			
Study selection	17	Give numbers of studies screened, assessed for eligibility, and included in the review, with reasons for exclusions at each stage, ideally with a flow diagram.	5
Study characteristics	18	For each study, present characteristics for which data were extracted (e.g., study size, PICOS, follow-up period) and provide the citations.	5
Risk of bias within studies	19	Present data on risk of bias of each study and, if available, any outcome level assessment (see item 12).	5
Results of individual studies	20	For all outcomes considered (benefits or harms), present, for each study: (a) simple summary data for each intervention group (b) effect estimates and confidence intervals, ideally with a forest plot.	5
Synthesis of results	21	Present results of each meta-analysis done, including confidence intervals and measures of consistency.	5
Risk of bias across studies	22	Present results of any assessment of risk of bias across studies (see Item 15).	6
Additional analysis	23	Give results of additional analyses, if done (e.g., sensitivity or subgroup analyses, meta-regression [see Item 16]).	6
**DISCUSSION**			
Summary of evidence	24	Summarize the main findings including the strength of evidence for each main outcome; consider their relevance to key groups (e.g., healthcare providers, users, and policy makers).	7
Limitations	25	Discuss limitations at study and outcome level (e.g., risk of bias), and at review-level (e.g., incomplete retrieval of identified research, reporting bias).	10
Conclusions	26	Provide a general interpretation of the results in the context of other evidence, and implications for future research.	10

**FUNDING**			
Funding	27	Describe sources of funding for the systematic review and other support (e.g., supply of data); role of funders for the systematic review.	11

Table S2. Univariate meta-regression of D-dimer and 36 demographic features. Cardiovascular disease includes cardiovascular disease, chronic heart failure, heart disease, peripheral vascular disease, cardiovascular and cerebrovascular, and atrial fibrillation. Chronic lung disease is composed of chronic lung diseases, respiratory system diseases, and tuberculosis. Cerebrovascular disease covers cerebrovascular disease, nervous system diseases, and cardiovascular and cerebrovascular diseases. Chronic liver disease consists of chronic liver disease, chronic hepatic/renal disease, and hepatitis B virus surface antigen positivity. Immunosuppression is a sum of immunosuppression and HIV infection. Chronic kidney disease is a combination of chronic kidney disease and chronic hepatic/renal disease. Autoimmune disease comprises autoimmune disease and rheumatic diseases.

**Variable**	**# of obs**	**Tau^2**	**I^2 (%)**	**Adj R^2 (%)**	**Slope coefficient (%)**	**T**	**P**	**95% CI**	**Intercept**
Age	80	.9373	97.10	24.06	.0425795 ± .0102776	4.14	<0.001	.0221184, .0630405	-1.303526 ± .5856676
Male (%)	78	.6706	97.22	22.79	.047403 ± .011614	4.08	<0.001	.0242718, .0705342	-1.90889 ± .7035323
Female (%)	78	.6706	97.22	22.79	-.047403 ± .011614	-4.08	<0.001	-.0705342, -.0242718	2.83141 ± .4811181
BMI	6	3.075	96.84	9.63	.3358127 ± .2808889	1.20	0.298	-.44406, 1.115685	-7.195167 ± 7.910486
Temperature	21	.1455	98.49	-7.55	-.0180809 ± .2039665	-0.09	0.930	-.4449878, .408826	1.179662 ± 7.792047
Heart rate	21	.19	98.22	-10.22	.0193019 ± .0335659	0.58	0.572	-.0509523, .0895561	-1.00516 ± 2.989607
Respiratory rate	19	.18	98.54	-1.43	.7493761 ± .227521	3.29	0.004	.2693488, 1.229403	-14.34925 ± 4.590821
Systolic pressure	21	.09	97.61	46.67	.0179975 ± .0054737	3.29	0.004	.0065409, .029454	-1.503423 ± .6321012
Diastolic pressure	6	.03	87.01	89.60	-.1546971 ± .041717	-3.71	0.021	-.2705219, -.0388722	12.79466 ± 3.223573
Smoking history	24	.08	95.54	-6.59	.0035268 ± .0082727	0.43	0.674	-.0136297, .0206833	.5236093 ± .1143706
Current smoker	19	.06	93.49	26.04	.0420279 ± .021866	1.92	0.072	-.0041072, .088163	.2708315 ± .1461845
Former smoker	6	.18	95.77	-12.50	-.1911086 ± .203683	-0.94	0.401	-.7566242, .374407	1.372812 ± .9031625

Fever	59	.2966	97.38	-5.41	.0013221 ± .0046383	0.29	0.777	-.007966, .0106103	.5507782 ±.3873433
Headache	37	.51	94.62	2.19	-.0189878 ± .0178216	-1.07	0.294	-.0551677, .0171921	.8741524 ± .249926
Dizziness	12	.25	97.45	-17.89	-.0075447 ± .041027	-0.18	0.858	-.0989606, .0838713	.7399351 ± .3692757
Dry cough	58	.2755	95.09	-3.86	-.0051107 ± .0048307	-1.06	0.295	-.0147878, .0045663	.9551633 ± .3172407
Fatigue	41	.5319	94.41	7.04	-.0103324 ± .008164	-1.27	0.213	-.0268455, .0061808	1.2704 ± .4385332
Myalgia	37	.42	95.24	12.61	-.0138942 ± .008413	-1.65	0.108	-.0309735, .0031851	1.083531 ± .2448052
Sputum	38	.6022	96.13	-8.76	-.0001632 ± .0152275	-0.01	0.992	-.0310459, .0307195	.8587777 ± .5665961
Dyspnoea/tachypnoea	45	.3166	95.66	23.35	.0187361 ± .0057624	3.25	0.002	.0071151, .0303571	.1034844 ± .2256114
Pharyngeal congestion	24	.8345	96.49	2.44	-.0431999 ± .0333224	-1.30	0.208	-.1123065, .0259066	1.239669 ± .3497917
Anorexia	19	1.52	97.16	2.13	.0226891 ± .0237709	0.95	0.353	-.0274632, .0728414	.3826393 ± .7857436
Vomiting/diarrhoea	49	.3214	94.80	1.92	.0176516 ± .01311	1.35	0.185	-.0087223, .0440255	.4532633 ± .1910005
Chest tightness	27	.76	96.19	0.50	-.0087195 ± .012162	-0.72	0.480	-.0337691, .0163301	1.022144 ± .3323422
Any comorbidity	45	.6215	97.68	14.57	.0197506 ± .0088202	2.24	0.030	.001963, .0375382	-.1167029 ± .4739545
Hypertension	60	.43	98.26	28.01	.0267183 ± .008342	3.20	0.002	.01002, .0434166	.0454089 ± .2700599
Diabetes	61	.3857	98.03	36.42	.0419345 ± .0121876	3.44	0.001	.0175472, .0663218	.1682089 ± .2199996
Chronic kidney disease	42	.7037	97.99	10.72	.094124 ± .0583258	1.61	0.114	-.0237569, .2120049	.6304573 ± .2345501
Chronic lung disease	62	.4652	98.55	20.92	.1035648 ± .029504	3.51	0.001	.044548, .1625816	.3895771 ± .1588404
Cerebrovascular disease	45	.6809	97.91	1.17	.0198492 ± .0169124	1.17	0.247	-.0142578, .0539563	.710586 ± .1938531
Cardiovascular disease	77	.6572	99.03	0.31	.0120193 ± .0105262	1.14	0.257	-.0089499, .0329886	.8300222 ± .1675696
Chronic liver disease	36	.8433	95.99	-2.76	-.0563075 ± .0605312	-0.93	0.359	-.1793218, .0667067	1.03306 ± .3224396

Malignancy	42	.80	98.78	4.06	.0083515 ± .007177	1.16	0.251	-.0061537, .0228567	.9381395 ± .1911616
Immunosuppression	15	.07893	98.54	-12.13	.0273784 ± .075834	0.36	0.724	-.136451, .1912078	.4244938 ± .1423138
Autoimmune disease	9	0.1469	95.02	43.14	-.6690673 ± .4543313	-1.47	0.184	-1.74339, .4052556	1.830882 ± .7610424
Endocrine system disease	7	.27	95.53	-22.05	.0176681 ± .0555458	0.32	0.763	-.1251169, .1604531	.4836994 ± .3450593

Table S3. Univariate meta-regression of D-dimer and sub-grouped age.

Variable	# of obs	Tau^2	I^2 (%)	Adj R^2 (%)	Slope coefficient (%)	T	P	95% CI	Intercept
Overall Age	80	0.9373	97.10	24.06	.0425795 ± .0102776	4.14	<0.001	.0221184, .0630405	-1.303526 ± .5856676
< 65	12	0.9977	93.28	56.79	-.0648731 ± .0229997	-2.82	0.018	-.1161195, -.0136266	5.588817 ± 1.576362
≥ 65	13	0.9818	92.72	57.81	.0673521 ± .0216528	3.11	0.010	.0196946, .1150096	-.9726664 ± .7955885
< 50	20	0.8618	99.04	24.10	-.0145913 ± .0067785	-2.15	0.045	-.0288324, -.0003502	1.60476 ± .4188022
≥ 50	19	0.8555	99.09	24.44	.015601 ± .0067145	2.32	0.033	.0014346, .0297674	.0725058 ± .4032568
< 60	13	0.07772	95.81	82.07	-.0449135 ±.0080865	-5.55	<0.001	-.0627117, -.0271154	3.157439 ± .4128981
≥ 60	13	0.0817	95.85	81.15	.0444832 ± .0082239	5.41	<0.001	.0263825, .062584	-1.307951 ± .4279083
≥ 70	7	0.1614	98.07	73.30	.0406418 ± .0106835	3.80	0.013	.0131789, .0681047	-.2746838 ± .3520958

Table S4. Univariate meta-regression of D-dimer and laboratory tests and radiology (62 variables in total).

Variable	# of obs	Tau^2	I^2 (%)	Adj R^2 (%)	Slope coefficient (%)	T	P	95% CI	Intercept
Platelet	64	1.33	97.18	18.65	.0139247 ± .0051553	2.70	0.009	.0036193, .0242301	-1.416173 ± 1.017723
PT	61	1.01	98.14	6.34	.0246973 ± .0160836	1.54	0.130	-.0074858, .0568805	.5974094 ± .4130279
INR	19	2.30	92.09	28.87	18.41005 ± 9.893327	1.86	0.080	-2.463043, 39.28315	-17.73264 ± 10.58759
APTT	52	1.95	97.57	-3.37	-.0110523 ± .0460678	-0.24	0.811	-.1035822, .0814776	1.769322 ± 1.520746
FDP	10	0.04	87.17	90.56	.2211652 ± .1000808	2.21	0.058	-.0096215, .451952	.087964 ± .2949227
Fibrinogen	27	1.83	92.90	45.94	1.076963 ± .3907774	2.76	0.011	.2721414, 1.881784	-3.488853 ± 1.963118
AT	8	23.73	76.70	-120.90	-.3764578 ± .494083	-0.76	0.475	-1.585435, .8325199	39.39957 ± 43.70739
PaO2/FiO2	14	0.12	85.94	-28.35	-.0021007 ± .0020121	-1.04	0.317	-.0064848, .0022833	1.255183 ± .5412732
PaO2	13	0.19	93.59	-7.28	-.0112423 ± .0099797	-1.13	0.284	-.0332075, .0107229	1.505157 ± .8579474
PaCO2	9	0.32	88.41	-48.66	-.0544023 ± .09302	-0.58	0.577	-.2743597, .165555	2.843023 ± 3.495582
SpO2	10	0.04	89.95	46.77	-.183205 ± .0520825	-3.52	0.008	-.3033075, -.0631026	18.22082 ± 5.065473
Lactate	9	0.42	99.02	-13.56	-.1061079 ± .3305823	-0.32	0.758	-.8878108, .6755951	1.159056 ± .7095926
Blood glucose	14	0.66	95.86	61.42	.9783473 ± .2679066	3.65	0.003	.3946291, 1.562066	-5.47596 ± 1.847775
WBC	71	0.24	97.32	65.26	.5181664 ± .0658182	7.87	<0.001	.3868627, .6494701	-2.232533 ± .3851643
Neutrophil	53	0.17	94.38	68.98	.4468458 ± .0694275	6.44	<0.001	.3074641, .5862274	-1.082089 ± .2869826
Monocyte	17	2.05	97.37	-9.06	-3.617222 ± 7.188717	-0.50	0.622	-18.93961, 11.70517	2.797381 ± 2.780371
Lymphocyte	70	0.63	98.26	7.70	-.6057299 ± .2552418	-2.37	0.020	-1.115057, -.0964028	1.481388 ± .29294
CD3+ T cell	10	0.00	56.27	-277.10	-.0009229 ± .000526	-1.75	0.117	-.0021359, .00029	1.183945 ± .3520576

CD4+ T cell	21	0.14	94.41	29.00	-.0041741 ± .0016122	-2.59	0.018	-.0075486, -.0007997	2.232315 ± .5385089
CD8+ T cell	21	0.05	93.02	75.33	-.0082453 ± .0017394	-4.74	<0.001	-.0118859, -.0046048	2.573746 ± .3855456
Hemoglobin	37	1.50	97.32	27.70	-.156854 .0439883	-3.57	0.001	-.246155 -.0675531	21.60028 5.672737
CK	51	0.24	97.22	47.26	.0060697 ± .0013804	4.40	<0.001	.0032956, .0088437	.0663016 ± .1691635
CK-MB	24	0.07	96.00	45.47	-.0320191 ± .0120122	-2.67	0.014	-.0569309, -.0071072	1.024705 ± .1817229
ALT	62	0.21	97.84	60.11	.0724162 ± .0120833	5.99	<0.001	.048246, .0965865	-1.190877 ± .3328446
AST	53	0.22	98.31	56.96	.0523305 ± .0087971	5.95	<0.001	.0346696, .0699913	-.9586733 ± .3011436
Total protein	5	0.09	71.11	-7235.92	-1.330099 ± .6196773	-2.15	0.121	-3.302188, .6419907	85.74198 ± 39.62798
Albumin	41	0.40	96.76	29.29	-.1575975 ± .0453491	-3.48	0.001	-.2493248, -.0658703	6.265189 ± 1.565779
Globulin	8	0.01	91.43	83.51	.1674196 ± .05055	3.31	0.016	.0437283, .291111	-4.539137 ± 1.477978
Prealbumin	5	0.68	77.67	-0.001	-.0254888 ± .0340792	-0.75	0.509	-.1339441, .0829664	3.863989 ± 3.918833
Total bilirubin	36	0.25	94.91	50.40	.2454894 ± .0528426	4.65	<0.001	.1381003, .3528786	-1.773512 ± .5511654
Direct bilirubin	10	0.01	84.47	-72.54	.011623 ± .0884742	0.13	0.899	-.1923989, .215645	.4919093 ± .3535077
Cr	58	0.63	96.25	-7.48	.0033323 ± .0071614	0.47	0.644	-.0110137, .0176783	.6391898 ± .4892071
BUN	31	.09	93.27	87.22	.651524 ± .0774167	8.42	<0.001	.4931891, .8098589	-2.378725 ± .3717765
eGFR	9	0.11	94.69	37.84	-.0450192 ± .0187385	-2.40	0.047	-.0893287, -.0007098	5.246897 ± 1.837197
LDH	51	0.20	98.10	62.65	.0070399 ± .0009898	7.11	<0.001	.0050509, .0090289	-1.306734 ± .3081648
Alkaline phosphatase	5	0.00	53.66	100.00	.0834717 ± .0130342	6.40	0.008	.041991, .1249524	-4.693745 ± .7946188
γ-glutamyl transpeptidase	8	0.07	93.81	73.76	.1930893 ± .0533137	3.62	0.011	.0626353, .3235433	-5.084917 ± 1.613093

BNP	13	0.15	91.75	-22.88	.0002438 ± .0007028	0.35	0.735	-.0013032, .0017907	.6877907 ± .2109536
Hypersensitive troponin I	19	0.13	96.19	-3.47	.0069077 ± .0056266	1.23	0.236	-.0049633, .0187787	.6561846 ± .1304793
Myoglobin	11	0.02	64.87	67.83	.0117508 ± .0032544	3.61	0.006	.004389, .0191127	.298485 ± .1157052
pH	5	0.21	88.43	-15.21	2.04637 ± 2.860102	0.72	0.526	-7.055751, 11.14849	-14.3333 ± 20.98662
K^+	16	0.12	93.30	64.07	2.186505 ± .5231928	4.18	0.001	1.064368, 3.308642	-7.976837 ± 2.096378
Na^+	17	0.34	98.47	-4.73	-.0998783 ± .161407	-0.62	0.545	-.4439093, .2441526	14.63029 ± 22.35994
PCT	53	.24	98.15	50.76	1.137369± .2077688	5.47	<0.001	.7202554, 1.554482	.551336± .0966591
CRP	45	0.43	98.14	48.18	.0133393 ± .0037963	3.51	0.001	.0056832, .0209954	.3523227 ± .1936306
hsCRP	15	0.13	88.93	-28.85	.009067 ± .0062126	1.46	0.168	-.0043544, .0224884	.3424013 ± .3446464
Ferritin	23	0.57	97.64	13.05	.0019119 ± .0006487	2.95	0.008	.0005628, .0032609	-.3499767 ± .5397798
ESR	17	0.03	91.93	42.93	.0089759 ± .0038022	2.36	0.032	.0008717, .0170801	.1878233 ± .1400137
IgA	7	0.07	83.83	-23.62	-.4928858 ± 1.398685	-0.35	0.739	-4.08832, 3.102549	1.909549 ± 3.216368
IgG	7	0.08	86.06	-29.16	-.0081512 ± .4538334	-0.02	0.986	-1.174767, 1.158465	.8754097 ± 5.293706
IgM	7	0.07	86.82	-15.22	-.6894745 ± 2.010037	-0.34	0.746	-5.856438, 4.477489	1.413422 ± 1.872564
IL2R	9	0.08	74.86	45.90	.0026641 ± .0010892	2.45	0.044	.0000886, .0052395	-1.001675 ± .7466923
IL6	28	0.32	95.28	-10.23	.0114648 ± .004989	2.30	0.030	.0012099,.0217198	.6870622 ± .1919376
IL10	9	0.39	94.71	-146.35	.190011 ± .1198651	1.59	0.157	-.0934248, .4734469	-.2125859 ± .7777158
IL8	9	0.09	83.03	45.39	.1220623 ± .0444439	2.75	0.029	.0169691, .2271554	-.9577867 ± .6515857
TNFα	9	0.14	71.16	9.65	.5433434 ± .2289427	2.37	0.049	.00198, 1.084707	-3.732038 ± 1.926771

Ground-glass opacity	22	1.03	93.53	-4.46	.0068567 ± .0092554	0.74	0.467	-.0124497, .0261632	.5731311 ± .6354694
Consolidation	12	0.14	91.28	8.01	.0095565 ± .0076037	1.26	0.237	-.0073856, .0264986	.4153754 ± .2162123
Local patchy opacities	10	2.18	97.19	1.56	-.088531 ± .0669269	-1.32	0.222	-.2428647, .0658026	2.352389 ± .9635898
Bilater alpatchy opacities	39	.36	94.33	-1.37	-.0045848 ± .0076426	-0.60	0.552	-.0200703, .0109006	1.020875± .6605744
Linear opacity	6	.261	89.29	-29.36	.0027267±.0201433	0.14	0.899	-.0532, .0586534	.7514164± .5327669
Pleural effusion	10	0.21	96.69	-14.57	-.0061905 ± .0297896	-0.21	0.841	-.0748853, .0625044	.5932003 ± .2669083

Table S5. Univariate meta-regression of fibrinolysis and life-saving supports and treatments. CRRT, continuous renal replacement therapy in total 11 variables). ECMO, extracorporeal membrane oxygenation. Heparin includes heparin, LMWH, and UFH. Antiviral therapy covers lopinavir-ritonavir, arbidol, oseltamivir, peramivir, ganciclovir, and ribavirin. Antibiotics comprises meropenem, impipenen/cilastatin, moxifloxacin, levofloxacin, cephalosporin, carbapenems, linezolid, vancomycin, teicoplanin, tigecycline, piperacillin/tazobactam, ceftriaxone sodium, cefoperazone/sulbactam, and ceftazidime tazobactam. Immune enhancement is a combination of immunoglobulin immune enhancer and thymalfasin. O2 treatment was pooled for oxygen, nasal cannula or no oxygen therapy, nasal cannula, high flow nasal cannula, and high-flow nasal cannula. Ventilation includes extracorporeal membrane oxygenation (ECMO), mechanical ventilation, non-invasive or invasive, prone position ventilation, non-invasive ventilation, non-invasive ventilation, and ECMO).

**Variable**	**# of obs**	**Tau^2**	**I^2 (%)**	**Adj R^2 (%)**	**Slope coefficient (%)**	**T**	**P**	**95% CI**	**Intercept**
Heparin	6	1.231	51.35	-197.84	.000783 ± .0364219	0.02	0.984	-.1003404, .1019065	2.236908 ± 1.283056
Immune enhancement	24	1.112	94.50	3.65	.0234749 ± .0129695	1.81	0.084	-.0034221, .050372	.3282648 ± .4130179
Interferon	17	1.514	93.38	-11.17	-.0040396 ± .0099133	-0.41	0.689	-.0251693, .0170901	.9207276 ± .5501961
Glucocorticoid	35	.6405	95.40	-4.81	.0073833 ± .0061798	1.19	0.241	-.0051896, .0199562	.5098028 ± .3230929
Antiviral therapy	42	.7045	95.28	-0.72	.0050323 ± .006825	0.74	0.465	-.0087615, .0188262	.5015258 ± .553464
Lopinavir-ritonavir (HIV)	11	.000086	91.33	95.86	.0027048 ± .0024193	1.12	0.293	-.0027682, .0081777	.1632012 ± .0500209
Antibiotics	33	.6862	91.82	4.58	.0122444 ± .0083186	1.47	0.151	-.0047216, .0292104	-.0692028 ± .699374
Antifungal	7	2.015	96.12	20.87	.1459324 ± .0932343	1.57	0.178	-.0937339, .3855987	.4912262 ± .7522512
O_2 therapy	49	.2647	95.05	-7.12	-.0007594 ± .0031547	-0.24	0.811	-.0071059, .005587	.7120668 ± .18288
CRRT	19	1.396	95.19	4.93	.0198778 ± .0183324	1.08	0.293	-.0188003, .0585558	1.038883 ± .3569355
Ventilation	71	.7513	96.11	28.50	.0188895 ± .0045525	4.15	<0.001	.0098075, .0279714	.5847255 ± .1574322

Table S6. Univariate meta-regression of D-dimer and 10 complications. ALI/ARDS is a combination of respiratory failure, ARDS, acute respiratory injury, pneumothorax, cardiopulmonary failure, ventilation associated pneumonia, any organ dysfunction, and multiple organ dysfunction syndrome. Acute cardiac injury includes acute cardiac injury, cardiopulmonary failure, acute coronary syndrome, heart failure, cardiac arrest, arrhythmia, any organ dysfunction, and multiple organ dysfunction syndrome. Secondary infection comprises secondary infection, hospital-acquired pneumonia, urinary tract infection, and ventilator-associated pneumonia. Brain injury is composed of hypoxic encehalopathy, cerebral ischemic attack, any organ dysfunction, and multiple organ dysfunction syndrome. Sepsis was combined with shock/septic shock and bacteraemia. Acute kidney injury was combined with any organ dysfunction, and multiple organ dysfunction syndrome. Coagulopathy includes coagulopathy, limb ischemia, mesenteric ischemia, cerebral ischemic attack, deep vein thrombosis, pulmonary embolism, ECMO oxygenator thrombosis, thrombo-embolic complications, gastrointestinal bleeding, and hemorrhagic complications. Acute liver injury covers acute kidney injury, any organ dysfunction, and multiple organ dysfunction syndrome. Disseminated intravascular coagulation (DIC).

**Variable**	**# of obs**	**Tau^2**	**I^2 (%)**	**Adj R^2 (%)**	**Slope coefficient (%)**	**T**	**P**	**95% CI**	**Intercept**
ALI/ARDS	48	.8132	95.29	56.63	.0333172 ± .0049013	6.80	<0.001	.0234514, .0431829	.1555065 ± .2201826
Acute cardiac injury	42	.4123	96.14	49.65	.048492 ± .0079245	6.12	<0.001	.0324759, .064508	.0867526 ± .1911167
Sepsis	34	.691	95.17	36.52	.0288675 ± .0068906	4.19	<0.001	.0148318, .0429033	.5330387 ± .2309609
Secondary infection	17	.01042	74.66	85.62	.0181947 ± .0031457	5.78	<0.001	.0114898, .0248997	.4638822 ± .0546524
Coagulopathy	17	1.452	90.37	38.05	.041048 ± .0141855	2.89	0.011	.0108125, .0712836	1.617012 ± .4105545
Acute kidney injury	28	.1824	95.79	-1.61	.0293776 ± .0111297	2.64	0.014	.0065002, .0522549	.5347102 ± .1514229
Acute liver injury	19	.9789	88.84	38.29	.05498 ± .0203131	2.71	0.015	.0121232, .0978368	.5502633 ± .4320377
DIC	6	.006059	65.90	95.63	.041824 ± .0148364	2.82	0.048	.0006314, .0830165	.6357128 ± .1074633
Brain injury	7	.119	80.57	29.32	.0760163 ± .0484944	1.57	0.178	-.0486425, .200675	.3769509 ± .3209676
Acidosis	6	.2965	83.30	-413.84	.1153982 ± .0687375	1.68	0.168	-.0754475, .306244	.020417 ± .6673867

Table S7. Univariate meta-regression of D-dimer and hospitalization as well as outcomes (10 variables in total).

**Variable**	**# of obs**	**Tau^2**	**I^2 (%)**	**Adj R^2 (%)**	**Slope coefficient (%)**	**T**	**P**	**95% CI**	**Intercept**
Days in hospital	24	.09564	93.88	16.89	.0186751 ± .0168438	1.11	0.280	-.0162568, .0536071	.3288056 ± .3052119
SOFA (sepsis)	6	5.138	96.67	-16.21	.2563246 ± .4375372	0.59	0.589	-.9584734, 1.471123	1.200765 ± 1.97019
CURB 65 (ARDS)	8	.3364	80.54	87.65	1.944863 ± .3816907	5.10	0.002	1.010899, 2.878826	.5629482 ± .270521
Onset to hospital	31	.4082	96.47	31.89	.1790362 ± .0641962	2.79	0.009	.0477401, .3103322	-.5856027 ± .4843322
Onset to dyspnea	11	.01197	86.03	69.35	.0895707 ± .0303624	2.95	0.016	.0208862, .1582552	-.1029336 ± .1827476
Onset to ARDS	7	3.629	94.41	5.65	.6305567 ± .4678449	1.35	0.236	-.5720767, 1.83319	-4.551236 ± 4.540552
Mortality	72	.5871	96.95	52.65	.0311312 ± .0045193	6.89	<0.001	.0221178, .0401446	.5566326 ± .1293366
Discharged	45	.1966	97.17	11.96	-.0068527 ± .0025594	-2.68	0.010	-.0120143, -.0016911	1.171189 ± .1863443
PCR negative time	8	0	36.50	100	.0350172 ± .0057767	6.06	0.001	.0208821, .0491523	-.0722968 ± .0710305
Hospitalized	23	.1371	97.54	5.96	-.0042131 ± .0031968	-1.32	0.202	-.0108613, .0024351	.8445181 ± .1776669

Table S8. Bivariate meta-regression of D-dimer and age.

Variable	Tau2	I^2 (%)	Adj R^2 (%)	Knapp-Hartung test	Age_Coef	Covariate_Coef	P_Age, P_Covariate
Male (%)	.4756	96.94	47.12	20.35, 0.0000	.0345558 ± .0076404	.045218 ± .010033	0.000, 0.000
Female (%)	.4756	96.94	47.12	20.35, 0.0000	.0345558 ± .0076404	-.045218 ± .010033	0.000, 0.000
Respiratory rate (/min)	.07294	95.90	59.84	14.25, 0.0003	.052489 ± .0126692	.5309503 ± .1901186	0.001, 0.013
Systolic pressure (mmHg)	.0334	95.86	79.10	21.04, 0.0000	.0274907 ± .0066139	.0109061 ± .003738	0.001, 0.009
Diastolic pressure (mmHg)	.03144	89.24	88.04	6.57, 0.0802	.0292335 ± .0310946	-.0647141 ± .105913	0.416, 0.584
Dyspnoea/tachypnoea (%)	.318	95.76	23.00	6.04, 0.005	.0122535 ± .0103823	.0147186 ± .0066839	0.245, 0.033
**Any comorbidity** (%)	.3875	96.22	46.36	10.09, 0.0003	.0834433 ± .0225037	-3.423298 ± .979832	0.001, 0.367
Hypertension (%)	.289	96.97	51.56	13.62, 0.0000	.0714525 ± .0189011	-.0040043 ± .0105522	0.000, 0.706
Diabetes (%)	.2748	96.90	54.29	14.70, 0.0000	.0566936 ± .0150699	.013864 ± .0128156	0.000, 0.284
Chronic lung diseases (%)	.3274	97.26	45.61	11.13, 0.0001	.0595491 ± .0163086	.0116849 ± .0341232	0.001, 0.733
Platelet (× 10^9/L)	.8667	96.73	47.17	18.08, 0.0000	.0878376 ± .0175372	.0092209 ± .0043626	0.000, 0.039
Fibrinogen (g/L)	1.772	90.64	44.55	5.86, 0.0116	.0886252 ± .0577845	.8994139 ± .3384015	0.143, 0.017
Serum K^+ (mM/L)	.01486	77.93	95.69	50.20, 0.0000	.0461923 ± .0082351	.3025944 ± .3577314	0.000, 0.413
WBC (× 10^9/L)	.07768	94.50	34.79	4.19, 0.0245	.0134585 ± .0048316	.0480968 ± .050246	0.009, 0.346
Neutrophil (× 10^9/L)	.146	94.48	72.74	23.45, 0.0000	.022279 ± .0114864	.3627258 ± .0749095	0.058, 0.000
Lymphocyte (× 10^9/L)	.5001	96.78	28.64	7.59, 0.0011	.0436423 ± .01372	.500105 ± .4674872	0.002, 0.289
CD4+ T cell (/µl)	.06289	94.25	66.43	10.84, 0.0011	.0440248 ± .0129318	-.0017927 ± .0012985	0.004, 0.186
CD8+ T cell (/µl)	.04375	93.86	76.65	13.54, 0.0004	.0274979 ± .0145424	-.004789 ± .0023011	0.077, 0.054
IL2R (U/mL)	.03677	76.22	76.53	4.86, 0.0557	.0580851 ± .039755	.000745 ± .0013745	0.194, 0.607

IL6 (pg/mL)	.174	87.29	34.58	8.50, 0.0017	.0610365 ± .0175608	.0063141 ± .0042264	0.002, 0.149
IL8 (pg/mL)	.03827	83.49	75.58	5.04, 0.0519	.053054 ± .0403638	.0438578 ± .0630927	0.237, 0.513
TNFα (pg/mL)	.03114	70.24	80.13	5.32, 0.0469	.0580475 ± .0320245	.1560514 ± .2212797	0.120, 0.507
Globulin (g/L)	.004372	81.58	90.86	10.23, 0.0171	.0166504 ± .0095824	.2052491 ± .0461679	0.143, 0.007
CRP (mg/L)	.5155	97.72	40.82	5.35, 0.0090	.0114784 ± .0110884	.0104171 ± .0049387	0.307, 0.042
Ferritin (μg/L)	.2444	97.62	62.54	6.66, 0.0061	.0587792 ± .0238059	.0010195 ± .000569	0.023, 0.088
ESR (mm/h)	.03978	79.70	35.91	2.58, 0.1110	-.0012875 ± .0060526	.0101547 ± .0063693	0.835, 0.133
LDH (U/L)	.184	97.25	66.13	27.58, 0.0000	.0149153 ± .013674	.0059977 ± .0013817	0.281, 0.000
PCT (ng/mL)	.1374	96.97	72.69	30.30, 0.0000	.0209681 ± .0045987	1.09963 ± .1754908	0.000, 0.000
SpO2 (%)	.06206	93.00	29.58	6.00, 0.0469	-.0066922 ± .0066224	-.2928946 ± .1040644	0.359, 0.037
Blood glucose (mM/L)	.5551	96.05	67.51	9.98, 0.0034	.0724658 ± .0372103	.6650597 ± .2818144	0.077, 0.038
eGFR (ml/min/1.73m^2)	.08806	94.21	51.19	4.22, 0.0628	.1014378 ± .0707111	.0131557 ± .04342	0.195, 0.771
Hemoglobin	1.133	96.66	45.34	12.80, 0.0001	.0919224 ± .0288498	-.048308 ± .0502507	0.003, 0.343
Albumin (g/L)	.2506	96.82	56.02	12.12, 0.0001	.0637003 ± .019848	-.0569807 ± .0456489	0.003, 0.220
BUN (mM/L)	.09901	92.73	86.19	37.65, 0.0000	-.0058244 ± .0049028	.725392 ± .0947993	0.245, 0.000
Total bilirubin (μM/L)	.2061	94.89	59.53	14.93, 0.0000	.0450554 ± .0178792	.1949147 ± .0507379	0.017, 0.001
Alkaline phosphatase (U/L)	0	62.20	100.00	16.95, 0.0577	.008795 ± .0131547	.0849493 ± .0146003	0.573, 0.028
GGT (U/L)	.07615	92.79	70.53	5.57, 0.0535	-.0039169 ± .0528515	.2011777 ± .1001843	0.944, 0.101
ALT (U/L)	.1952	96.88	62.40	20.40, 0.0000	.0119359 ± .0062124	.0614013 ± .0127305	0.060, 0.000
AST (U/L)	.1882	97.49	62.31	23.50, 0.0000	.0130397 ± .0056086	.0455549 ± .0086817	0.024, 0.000
Myoglobin (ng/mL)	.01958	68.45	62.23	6.02, 0.0254	-.0026532 ± .0390157	.0124321 ± .007751	0.947, 0.147

CK (U/L)	.1951	97.31	59.48	14.13, 0.0000	.0155365 ± .0061076	.0051129 ± .0013181	0.014, 0.000
CK-MB (U/L)	.07414	96.41	40.35	3.80, 0.0409	.0087226 ± .0055735	-.0151021 ± .0159855	0.134, 0.357
Mortality (%)	.5728	97.00	55.22	24.98, 0.0000	.0192994 ± .0091921	.0260759 ± .005086	0.040, 0.000
Ventilation (%)	.6692	96.29	48.27	14.82, 0.0000	.0563806 ± .0343562	.0165375 ± .0072555	0.109, 0.029
CURB 65 (lung)	.9307	97.11	24.60	9.82, 0.0002	.0450605 ± .0103717	-.0005064 ± .0003269	0.000, 0.126
Onset to dyspnea (day)	.01361	73.97	65.17	3.65, 0.0746	-.0026832 ± .0133044	.0901126 ± .0333361	0.845, 0.027
Onset to admission (day)	.3664	96.30	38.87	5.53, 0.0095	.0400727 ± .023766	.0766912 ± .084454	0.103, 0.372
Days for PCR-negative	0	63.77	100.00	9.38, 0.0512	-.0062251 ± .0145224	.0609907 ± .0630725	0.697, 0.405
Discharged (%)	.1475	96.95	39.08	7.85, 0.0015	.0180682 ± .0056076	-.0020336 ± .0027367	0.003, 0.462
ALI/ARDS (%)	.7651	95.84	61.62	19.60, 0.0000	.0480813 ± .0248756	.0256447 ± .0067261	0.063, 0.001
Secondary infection (%)	.01126	75.69	84.47	17.44, 0.0002	.021446 ± .0182595	.0087511 ± .0086295	0.260, 0.328
Sepsis (%)	.5282	94.58	56.89	12.20, 0.0003	.075812 ± .0259446	.0211361 ± .0068359	0.008, 0.006
Acute cardiac injury (%)	.3689	94.81	69.89	18.60, 0.0000	.0240925 ± .0255873	.046869 ± .010617	0.357, 0.000
Coagulopathy	.8606	91.32	78.63	12.98, 0.0044	.2263076 ± .0662787	.0168365 ± .01493	0.011, 0.297
Acute kidney injury (%)	.1221	94.94	21.70	5.47, 0.0123	.0373226 ± .0178561	.0155371 ± .0140996	0.049, 0.283
DIC (%)	0	63.19	100.00	5.47, 0.0998	.0224815 ± .0195806	.02901 ± .0174885	0.334, 0.196
Acute liver injury (%)	1.156	90.58	34.91	4.40, 0.0347	.0907047 ± .0607898	.0310989 ± .0275352	0.160, 0.279

Table S9. Bivariate meta-regression of D-dimer and male.

**Variable**	**Tau2**	**I^2 (%)**	**Adj R^2 (%)**	**Knapp-Hartung test**	**Male_Coef**	**Covariate_Coef**	**P_Age, P_Covariate**
Age (%)	.4756	96.94	47.12	20.35, 0.0000	.045218 ± .010033	.0345558 ± .0076404	0.000, 0.000
Respiratory rate (/min)	.1657	98.62	8.76	7.35, 0.0054	-.0253 ± .0140346	.8859835 ± .2328113	0.090, 0.003
Systolic pressure (mmHg)	.08106	97.68	49.28	5.79, 0.0114	-.0094118 ± .0107654	.0184609 ± .0054839	0.393, 0.003
Diastolic pressure (mmHg)	.03436	89.75	86.92	4.71, 0.1186	-.0105939 ± .0307118	-.1553723 ± .0509539	0.753, 0.055
Dyspnoea/tachypnoea (%)	.3398	95.63	17.74	5.30, 0.0089	.0036698 ± .0129002	.0185033 ± .0062514	0.777, 0.005
**Any comorbidity** (%)	.5543	96.56	23.26	5.20, 0.0096	.0348914 ± .0156788	.0173814 ± .0083171	**0.031, 0.043**
Hypertension (%)	.3859	97.31	35.33	8.29, 0.0007	.0293565 ± .0125173	.0268093 ± .007907	0.023, 0.001
Diabetes (%)	.4023	97.02	33.09	6.96, 0.0019	.0189905 ± .013134	.0366433 ± .0126802	0.154, 0.005
Chronic lung diseases (%)	.4892	97.58	18.72	6.23, 0.0038	.0359004 ± .0172547	.0665802 ± .0338718	0.043, 0.055
Platelet (× 10^9/L)	.7939	96.96	35.87	8.65, 0.0005	.0540378 ± .0143918	.0098261 ± .0052891	0.000, 0.068
Fibrinogen (g/L)	1.66	92.13	42.64	5.39, 0.0172	.0792087 ± .0391209	.0911898 ± .5338312	0.061, 0.867
Serum K^+ (mM/L)	.1198	93.25	65.25	8.74, 0.0039	-.0162702 ± .0224582	2.347222 ± .5773524	0.482, 0.001
WBC (× 10^9/L)	.125	94.92	-10.92	0.22, 0.8035	-.0022819 ± .0118263	.0431904 ± .0650708	0.848, 0.511
Neutrophil (× 10^9/L)	.1437	94.50	73.17	22.71, 0.0000	-.0168235 ± .0096665	.4943481 ± .0755642	0.088, 0.000
Lymphocyte (× 10^9/L)	.4044	97.36	9.21	4.44, 0.0157	.0239585 ± .0122158	-.5 ± .2124348	0.054, 0.022
CD4+ T cell (/µl)	.07305	94.75	61.01	9.00, 0.0024	-.0282222 ± .0094947	-.0054255 ± .0013644	0.009, 0.001
CD8+ T cell (/µl)	.04924	93.89	73.72	11.54, 0.0008	-.0106369 ± .0076828	-.0077193 ± .0017203	0.185, 0.000
IL2R (U/mL)	.1244	78.34	20.61	3.06, 0.1215	-.0114252 ± .0172269	.0029001 ± .0012234	0.532, 0.055
IL6 (pg/mL)	.3353	95.48	-26.04	2.48, 0.1060	.0009617 ± .0166648	.0112702 ± .0055427	0.954, 0.054

IL8 (pg/mL)	.004064	45.53	97.41	16.51, 0.0036	-.0166125 ± .0056893	.1095546 ± .0230553	0.027, 0.003
TNFα (pg/mL)	.1155	73.07	26.26	3.98, 0.0794	-.022454 ± .0161879	.5857414 ± .2160851	0.215, 0.035
Globulin (g/L)	.004425	78.05	90.75	6.76, 0.0378	.0053343 ± .005792	.1478049 ± .0477136	0.399, 0.027
CRP (mg/L)	.2688	97.52	57.54	9.58, 0.0004	.0139449 ± .0148991	.0124845 ± .0033627	0.355, 0.001
Ferritin (μg/L)	.27	97.41	8.81	3.27, 0.0602	-.050019 ± .0282147	.001909 ± .0007467	0.092, 0.019
ESR (mm/h)	.03948	90.74	36.38	2.67, 0.1042	.0037538 ± .0091448	.0087382 ± .004094	0.688, 0.051
LDH (U/L)	.18	97.28	66.87	29.20, 0.0000	-.0169912 ± .0100969	.007727 ± .0010275	0.099, 0.000
PCT (ng/mL)	.2387	97.27	50.10	15.03, 0.0000	.0023234 ± .0115448	1.123124 ± .210136	0.841, 0.000
SpO2 (%)	.04016	89.77	40.17	6.63, 0.0242	.0144281 ± .0168845	-.1922934 ± .0532755	0.421, 0.009
Blood glucose (mM/L)	.6818	95.81	60.10	6.97, 0.0111	-.0313233 ± .0350607	1.167446 ± .3411115	0.391, 0.006
eGFR (ml/min/1.73m^2)	.06348	91.44	64.81	5.12, 0.0427	.0433164 ± .0240815	-.0486565 ± .016176	0.115, 0.020
Hemoglobin	.6758	96.62	61.23	18.04, 0.0000	.0741141 ± .0166194	-.1561793 ± .03265	0.000, 0.000
Albumin (g/L)	.4289	96.70	24.73	6.01, 0.0054	.0049925 ± .0162472	-.1522545 ± .0528149	0.760, 0.006
BUN (mM/L)	.09092	93.11	87.31	36.21, 0.0000	-.0088647 ± .0080009	.6674104 ± .0785694	0.277, 0.000
Total bilirubin (μM/L)	.2627	94.83	48.41	10.97, 0.0002	-.0093571 ± .0137379	.2706163 ± .063173	0.501, 0.000
Alkaline phosphatase (U/L)	.003422	64.66	99.38	11.70, 0.0787	.0196495 ± .0283818	.0719833 ± .0267932	0.560, 0.115
GGT (U/L)	.07559	77.29	70.75	6.21, 0.0442	.0289868 ± .0454426	.1640931 ± .074286	0.552, 0.078
ALT (U/L)	.2044	96.89	60.63	17.88, 0.0000	-.0074919 ± .010546	.0751675 ± .0130881	0.480, 0.000
AST (U/L)	.212	97.55	57.53	19.73, 0.0000	-.0091311 ± .0107331	.0556312 ± .0089842	0.399, 0.000
Myoglobin (ng/mL)	.01892	68.57	63.50	6.46, 0.0214	.007426 ± .0118532	.0116007 ± .0034044	0.548, 0.009
CK (U/L)	.2545	97.22	43.96	9.71, 0.0003	.0079922 ± .0125197	.0059379 ± .001431	0.526, 0.000

CK-MB (U/L)	.0719	93.76	40.79	3.50, 0.0489	.0081901 ± .0140915	-.0379235 ± .0159903	0.567, 0.027
Mortality (%)	.4478	97.05	56.35	28.31, 0.0000	.0286637 ± .0105698	.0262888 .0042898	0.009, 0.000
Ventilation (%)	.649	96.40	49.84	14.17, 0.0000	.0241306 ± .0170254	.0216184 ± .0055418	0.165, 0.000
CURB 65 (lung)	.4719	80.39	82.67	11.65, 0.0131	-.0307255 ± .1001837	2.218864 ± .8838119	0.771, 0.054
Onset to dyspnea (day)	.0155	83.82	60.32	3.41, 0.0849	-.0028779 ± .0129826	.0996771 ± .0568761	0.830, 0.118
Onset to admission (day)	.2987	96.49	50.16	8.36, 0.0014	.0407723 ± .0149951	.1712355 ± .0549147	0.011, 0.004
Days for PCR-negative	0	25.14	100.00	22.70, 0.0031	.0096266 ± .0066849	.0376591 ± .0056277	0.209, 0.001
Discharged (%)	.2301	97.50	3.35	4.24, 0.0218	.0170597 ± .011718	-.0067567 ± .0027708	0.154, 0.020
ALI/ARDS (%)	.8654	96.06	56.59	16.60, 0.0000	.007648 ± .0207412	.0317132 ± .0073734	0.715, 0.000
Secondary infection (%)	.01029	66.71	85.81	16.29, 0.0002	-.0037729 ± .006143	.0167955 ± .0039349	0.549, 0.001
Sepsis (%)	.8009	96.07	34.64	6.52, 0.0063	.010374 ± .0229234	.027864 ± .0080554	0.656, 0.002
Acute cardiac injury (%)	.395	95.38	67.77	17.73, 0.0000	-.0030599 ± .0160666	.0534294 ± .0090987	0.851, 0.000
Coagulopathy	.09094	84.01	97.54	20.66, 0.0038	.1441181 ± .0268304	-.0151524 ± .0275977	0.003, 0.607
Acute kidney injury (%)	.1771	96.37	-13.53	3.76, 0.0402	-.0096154 ± .0131288	.0360883 ± .0136482	0.472, 0.015
DIC (%)	.2577	74.16	-85.97	3.16, 0.1825	.0646835 ± .051906	.0583958 ± .0289456	0.301, 0.137
Acute liver injury (%)	1.093	89.39	38.45	4.19, 0.0394	.0420395 ± .0302996	.0616988 ± .0223492	0.189, 0.016
